# Characterization of lignin-degrading enzyme PmdC, which catalyzes a key step in the synthesis of polymer precursor 2-pyrone-4,6-dicarboxylic acid

**DOI:** 10.1016/j.jbc.2024.107736

**Published:** 2024-08-31

**Authors:** Andria V. Rodrigues, Nigel W. Moriarty, Ramu Kakumanu, Andy DeGiovanni, Jose Henrique Pereira, Jennifer W. Gin, Yan Chen, Edward E.K. Baidoo, Christopher J. Petzold, Paul D. Adams

**Affiliations:** 1 Joint BioEnergy Institute, Emeryville, California, United States; 2 Molecular Biophysics and Integrated Bioimaging, Lawrence Berkeley National Laboratory, Berkeley, California, United States; 3 Biological Systems and Engineering Division, Lawrence Berkeley National Laboratory, Berkeley, California, United States; 4 Department of Energy Agile BioFoundry, Emeryville, California, United States; 5 Department of Bioengineering, University of California Berkeley, Berkeley, California, United States

**Keywords:** NADP oxidoreductase, lignin degradation, polymer synthesis, pyrone 2,4 dicarboxylic acid, enzyme catalysis, structure-function, crystal structure, alphafold, ligand docking, computational modeling

## Abstract

Pyrone-2,4-dicarboxylic acid (PDC) is a valuable polymer precursor that can be derived from the microbial degradation of lignin. The key enzyme in the microbial production of PDC is 4-carboxy-2-hydroxymuconate-6-semialdehyde (CHMS) dehydrogenase, which acts on the substrate CHMS. We present the crystal structure of CHMS dehydrogenase (PmdC from *Comamonas testosteroni*) bound to the cofactor NADP, shedding light on its three-dimensional architecture, and revealing residues responsible for binding NADP. Using a combination of structural homology, molecular docking, and quantum chemistry calculations, we have predicted the binding site of CHMS. Key histidine residues in a conserved sequence are identified as crucial for binding the hydroxyl group of CHMS and facilitating dehydrogenation with NADP. Mutating these histidine residues results in a loss of enzyme activity, leading to a proposed model for the enzyme’s mechanism. These findings are expected to help guide efforts in protein and metabolic engineering to enhance PDC yields in biological routes to polymer feedstock synthesis.

Efficient utilization of plant biomass, particularly lignin, is crucial to achieve the transition from a nonrenewable fossil-fuel–dependent economy to a renewable bio-based economy (1). Lignin is one of the most abundant raw materials available on earth (1, 2). Composed of a three-dimensional network of polymerized aromatic monomers, it is a major contributor to the rigidity of plant tissues. However, owing to its complex structure, it is naturally resistant to deconstruction and therefore largely underutilized (3). Unused lignin generated from pulping processes and second-generation biofuel refineries is predominantly burned for energy. However, recently, the potential of lignin as a raw material and carbon source for the manufacture of several high-value products has been recognized (1).

Depolymerization of lignin into low molecular weight aromatics employs a combination of physico-chemical and enzymatic methods (3). Aromatics released from lignin after depolymerization can be utilized by microbes possessing metabolic pathways that convert these aromatics into key metabolic intermediates such as 3-methyl gallate, protocatechuate, and vanillate. This is followed by aromatic ring cleavage into smaller molecules that can enter into central metabolic pathways (1). Many of these pathways have been engineered to produce high value products such as vanillin (4) or chemical feedstocks that can be used to synthesize polymers such as adipic acid (5), *cis*, *cis*-muconic acid (6), and pyrone-2, 4-dicarboxylic acid (PDC) (7). The term “lignin valorization” is commonly used to describe the synthesis of high value products from lignin. This process not only provides products that are sustainable alternatives to fossil fuels but also aids in economizing biofuels produced in second-generation refineries (1).

PDC is one such high-value product derived from the microbial degradation of lignin. PDC is structurally similar to terepthalic acid (TPA), a petroleum-derived chemical feedstock used in the synthesis of polymers such as polyethylene terephthalate. The pseudoaromatic ring of PDC, together with its two carboxylic groups, enables it to be used as a substitute for TPA (8). PDC has already been incorporated into the synthesis of polymers with several industrially desirable properties including thermostability, enhanced biodegradability, metal chelation, and strong adhesives to name a few (8, 9). In microbes, PDC is primarily generated by the breakdown of protocatechuate *via* the protocatechuate-4,5-cleavage pathway. This pathway has been best characterized in the bacterium *Sphingobium* strain SYK6. Here, ring cleavage of protocatechuate is catalyzed by an iron-dependent enzyme, protocatechuate-4,5-dioxygenase (LigAB) producing 4-carboxy-2-hydroxymuconate-6-semialdehyde (CHMS) with the incorporation of a molecule of oxygen. This is followed by the conversion of CHMS to PDC, catalyzed by CHMS dehydrogenase (LigC) (Fig. 1), (10).

**Figure 1:**
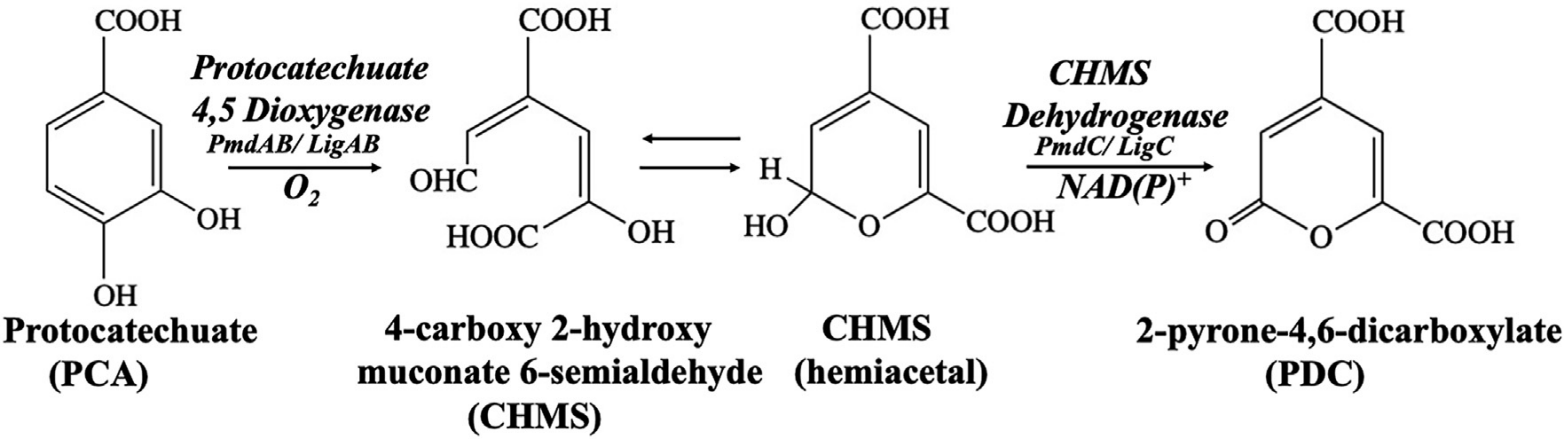
**Bacterial protocatechuate 4,5 cleavage pathway leading to the conversion of protocatechuate to polymer precursor 2-pyrone-4,6-dicarboxylate.** The breakdown of protocatechuate is catalyzed by iron-dependent enzyme Protocatechuate 4,5 dioxygenase (known as LigAB in *Sphingomonas* SYK-6 or PmdAB in *Comamonas testosteroni*) producing 4-carboxy 2-hydroxymuconate 6-semialdehyde (CHMS). CHMS exists in equilibrium between the open-chain and hemiacetal forms. The conversion of CHMS to PDC is catalyzed by CHMS dehydrogenase (known as LigC in *Sphingomonas* SYK-6 or PmdC in *Comamonas testosteroni*) *via* a reaction requiring NAD(P)^+^.

While chemical synthesis of PDC has been demonstrated, large-scale synthesis has yet to be performed (11). On the other hand, *in vivo* PDC production *via* metabolically engineered hosts has been much more successful and has been demonstrated in *Escherichia coli* (12, 13), *Pseudomonas putida* (7, 14, 15), *Novosphingobium aromaticivorans* (2), and *in planta* (9). PDC production has been demonstrated using a variety of aromatics, namely, aromatic monomers such as protocatechuate (13, 16), 4-hydroxybenzoate (17), p-coumarate (7), chemically depolymerized lignin containing a mixture of S, G, and H lignin aromatics (2), extracts from kraft lignin, softwood, and hardwood extracts (15), and from TPA recovered from polyethylene terephthalate recycling (8). These studies demonstrated the potential of using lignin-derived biomass and plastic waste towards the production of a high-value end product. In these engineered hosts, *ligABC* genes from *Sphingomonas* SYK-6 and *pmdABC* genes from *Comamonas testosteroni* were most frequently incorporated for PDC production. Besides these genes, Zhou *et al*. tested an additional 16 homologs of the LigABC type proteins obtained from BLAST analysis from among different bacterial classes which included Alphaproteobacteria, Betaproteobacteria, Gammaproteobacteria, and Actinobacteria. While two Actinobacteria-derived homologs showed faster accumulation of PDC than others, the conversion was however incomplete (18). Much effort is ongoing to improve PDC production by engineering host genetic pathways to improve titer by reducing flux toward competing pathways (2), incorporating protein homologs with desired activity rates (18) and enhancing the ability of microbial hosts to tolerate toxicity arising from high concentrations of aromatics (14).

Among the Protocatechuate, 4,5 dioxygenases LigAB (*Sphingomonas* SYK-6) and PmdAB (*C*. *testosteroni*) have been best characterized both structurally and functionally (19, 20, 21). The enzyme belongs to the family of extradiol-type catecholic dioxygenases that cleave the aromatic ring adjacent to the hydroxyl groups of protocatechuate (3,4 – dihydroxybenzoic acid) (19). The enzyme exists as an α_2_β_2_ heterotetramer with a nonheme ferrous iron center and, therefore, exhibits higher enzymatic activity when purified anaerobically under conditions where the metal center is maintained in the reduced state (21). CHMS dehydrogenase, on the other hand, has only been characterized functionally from *Sphingomonas* SYK-6 (10) and *Pseudomonas ochracea* (22). These studies have demonstrated that the enzyme is a NADP^+^/NAD^+^-dependent oxidoreductase with a cofactor preference for NADP^+^. In this study, we present the first known structural characterization of a CHMS dehydrogenase, PmdC from *C*. *testosteroni*. This structure bound to cofactor NADP was solved to a resolution of 2.34 Å. Activity assays reveal that the protein can use both NADP or NAD at nonlimiting cofactor concentrations. Based on a combination of three-dimensional structural homology, electron density, molecular docking, and quantum chemistry calculations, we identified the potential binding site of substrate CHMS and key residues involved in substrate binding. The substrate binding site has three histidine residues that potentially play a role in catalysis and are part of a highly conserved sequence ^168^RSWTDHLLWHHXXHXXDXF^186^ representative of the binding site. The consensus sequence ^157^E(L/A)HHXXKXDAPSGTA^171^ (*E*. *coli* numbering) containing two histidine and a lysine residue represents the binding site of dihydrodipicolinate to the dihydrodipicolinate reductase (DHDPR) enzyme, a substrate with a structure analogous to CHMS (23). In DHDPR, the histidine and lysine residues play important roles in substrate stabilization during catalysis. In PmdC, site-directed mutagenesis of the histidine residues resulted in loss of activity, indicating that the residues are most likely involved in catalysis. Using both biochemistry data and quantum calculations, we propose an acid-base catalysis mechanism mediated by the histidine residues facilitating hydride transfer to the nicotinamide ring and product stabilization, producing PDC. This study provides molecular level insight into cofactor and substrate binding and catalysis by a CHMS dehydrogenase and will aid metabolic engineering as well as enzyme engineering efforts for tunable enzyme activity.

## Results

### Activity assays monitoring enzymatic production of CHMS and conversion to PDC by PmdAB and PmdC

#### UV-Vis–based kinetics by PmdAB and PmdC

**Figure 2:**
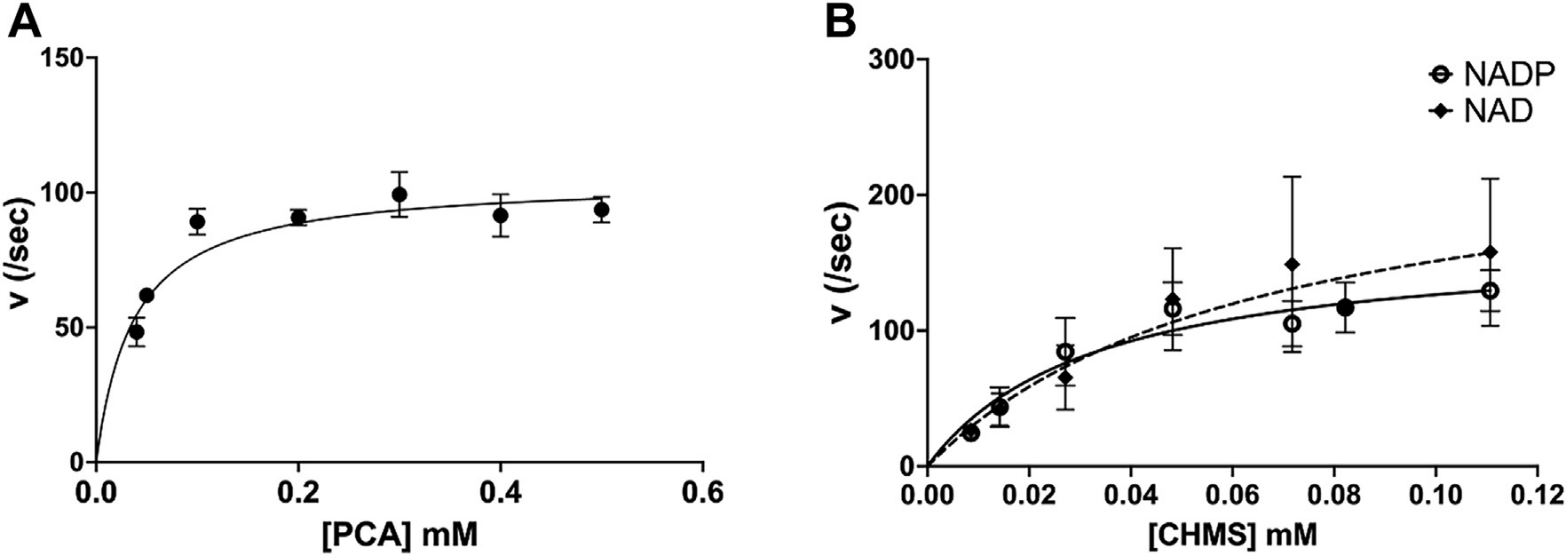
**Kinetic studies of the conversion of Protocatechuate to CHMS by PmdAB and CHMS to PDC by PmdC.** Michaelis-Menten plots of velocity (/sec) versus substrate concentration (mM) for the conversion of (*A*) Protocatechuate to CHMS by PmdAB and (*B*) CHMS to PDC by PmdC in the presence of cofactor NADP or NAD. Each point represents the mean and SD from three independent experiments.

Activity assays on CHMS dehydrogenases have been previously performed on homologs from *P*. *ochracea* (22) and *Sphinogomonas paucimobilis* SYK-6 (10). In these studies, the pH optimum for CHMS dehydrogenase activity was found to be between pH 7.0 to 8.0 at a temperature of 24 to 25 °C. Both methods utilized aerobically purified protocatechuate 4,5 dioxygenase for CHMS production. A marked decrease in activity was observed with aerobically purified dioxygenase LigAB in comparison to the anaerobically purified protein (19). Loss of activity of protocatechuate 4,5 dioxygenases is attributed to oxidation of the catalytic ferrous iron required for activity. To determine the kinetics of PmdC, the conversion of protocatechuic acid (PCA) to CHMS was first monitored using anaerobically purified PmdAB *via* UV-Visible spectrophotometry. Accumulation of a characteristic yellow product with an absorbance peak at 410 nm was observed corresponding to CHMS (19, 22). The intensity of this product was enhanced upon the addition of 3N NaOH (Fig. S1). The concentration of the yellow product was calculated using the known molar extinction coefficient of CHMS in the presence of NaOH, 29,000 M^−1^ cm^−1^ (24). By determining the absorbance in the absence of and upon adding NaOH, the molar extinction coefficient of CHMS in was determined to be 2547 M^−1^ cm^−1^, in good agreement with previously reported values (22). Equilibrium kinetics parameters for the conversion of PCA to CHMS were determined by monitoring the rise in absorbance at 410 nm at pH 7.5 and ambient temperature. Using the raw data of product accumulation versus time (Fig. S2*A*), the slope versus substrate concentration was plotted and the resulting curve was fit with a Michaelis Menten equation (Fig. 2*A*). Kinetics parameters are listed in Table 1. The PmdAB K_m_^app^ for PCA was found to be 33 ± 12 *μ*M with a K_cat_^app^ of 107.30 ± 7.5 s^-1^ and K_cat_^app^/K_m_^app^ of 3.25 × 10^6^ M^-1^ sec^-1^ comparable to anaerobically purified LigAB activity (19). The reported activity of aerobically purified enzyme from *P*. *ochracea* measured similarly was approximately three times lower than PmdAB (Table S3). For PmdC, kinetics parameters were determined by measuring the decrease in absorbance at 410 nm upon the addition of NADP/NAD and PmdC to the PmdAB reaction (Figs. 2*B* and S2, *B* and *C*). The K_m_^app^ of PmdC with NADP was 68.76 *μ*M ± 12 with a K_cat_^app^ of 37.51 ± 8.02 s^−1^; these parameters were similar for NAD, using which the K_m_^app^ was 62.70 ± 6.5 *μ*M and K_cat_^app^ was 35.89 ± 14.05 s^−1^. The K_cat_^app^/Km^app^ with NADP was 4.0 × 10^6^ and was similar using NAD which was 4.2 × 10^6^ (Table 1). The enzyme velocity of PmdC is comparable to that of LigC CHMS dehydrogenases when measured with a constant cofactor concentration at excess amounts of 1 mM NADP or NAD (Table S3). A control reaction containing PmdC without cofactor showed no activity (Fig. S2*D*). In previous studies, the reported activities of LigC from *S*. *paucimobilis* SYK-6 and *P*. *ochracea* were similar in the presence of a constant CHMS concentration, while the cofactor concentration was varied. A comparison of enzyme velocities of PmdC with LigC CHMS dehydrogenases from *S*. *paucimobilis* SYK-6 and *P*. *ochracea* are shown in Table S3. Using this method, the K_m_ for NADP was approximately 10 to 16 times lower than the K_m_ for NAD. However, when the cofactor concentration was kept constant with a varied CHMS concentration, the enzyme velocity was 2 times higher in the presence of NADP over NAD while Km for CHMS remained low with either cofactor (10, 22). It is to be noted that in both cases, assays where the cofactor concentration was constant were performed at an NAD concentration lower than the Km, which could account for enzyme velocity being under Vmax. A preference for NADP over NAD is plausible as evidenced by amino acid side-chain stabilization of the phosphate group of NADP in the crystal structure of PmdC (*vide infra*).

**Table 1:** PmdC & PmdAB kinetics parameters measured at 410 nm

Protein	Substrate	Km (μM)	kcat (s^−1^)	kcat/Km (M^−1^ s^−1^)
PmdC	CHMS, NADP	68.76 ± 2.4	277.55 ± 59.5	4.0 × 10^6^
	CHMS, NAD	62.70 ± 6.5	265.55 ± 104.0	4.2 × 10^6^
PmdAB	PCA	33.00 ± 12.0	107.3 ± 7.5	3.3 × 10^6^
Lig AB^a^	PCA	51.00 ± 4.0	216.0 ± 3.0	4.3 × 10^6^

a Barry KP *et al*., Biochemistry 2013.

#### LC-MS confirmation of the mass of enzymatically produced products by PmdAB and PmdC

The molecular masses of enzymatically produced CHMS and PDC from PCA catalyzed by PmdAB and PmdC were confirmed using LC-MS. Retention time and mass spectra of elution peaks corresponding to substrate PCA and product PDC were confirmed using pure standards. Pure standards for CHMS were unavailable; instead, the identity of CHMS was confirmed by LC/MS-MS alone. PCA eluted as a single sharp peak at a retention time of 6.30 min. The theoretical mass of PCA was 153.019332 *m/z*, while the observed mass was 153.019518 *m/z* for the standard and 153.019409 *m/z* in the experimental sample (Fig. S3*A*). PDC eluted at a retention time of 4.25 min. The theoretical mass of PDC was 182.993511 *m/z*, while the observed mass was 182.993555 *m/z* for the standard and 182.993063 *m/z* in the experimental sample (Fig. S3*B*). CHMS eluted as two separate peaks at retention times of 4.66 and 5.60 min. For CHMS, the theoretical mass was 185.009161 *m/z*, the observed mass for the earlier peak was 185.009504 *m/z*, while that of the later peak was 185.009597 *m/z* (Fig. S3*C*). The observed mass error for all analytes was ≤2.5 ppm, which is indicative of good-to-excellent mass accuracy.

While LC-MS confirmed the enzymatic production of the expected products, that is, CHMS by PmdAB and PDC by PmdC, the nature of the two CHMS elution peaks were further investigated. Both peaks yielded a similar mass spectrum by LC/MS-MS, indicating they might be two different forms of the same CHMS molecule. Experimentally, the major fragment ions observed were at 41.040318 m/z, 67.019049 m/z, and 69.035170 m/z for peak 1 and 41.039830 m/z, 67.018609 m/z, and 69.034861 m/z for peak 2 (Fig. S3*D*, panels A and B). To investigate whether the two chromatographic peaks may correspond to the open-chain and hemiacetal forms of CHMS, the fragmentation pattern was analyzed by calculating the mass of fragments formed by systematically cleaving each bond. Fragments with masses identical to the experimentally observed ions were assigned. A similar fragmentation pattern was obtained with both the hemiacetal and open-chain forms of CHMS (Fig. S3*D*, panel C). Based on these results, we speculate that the two peaks could likely represent each form of CHMS or each peak could be a mixture of the two forms differing in pKa due to differences in protonation of the carboxylic acid groups. It is also highly likely that the different forms of CHMS are converted into a single species (*e*.*g*., [M-H]^-^) at the ionization source. Additionally, since the samples were run on a reversed phase chromatography column as opposed to a chiral column, it is unlikely that the two peaks correspond to stereoisomers of CHMS differing at the C2 position, that is, (R)- and (S)-2-hydroxy-2*H*-pyran-4,6-dicarboxylic acid.

### Crystal structure of PmdC–NADP with modeled CHMS substrate

**Figure 3:**
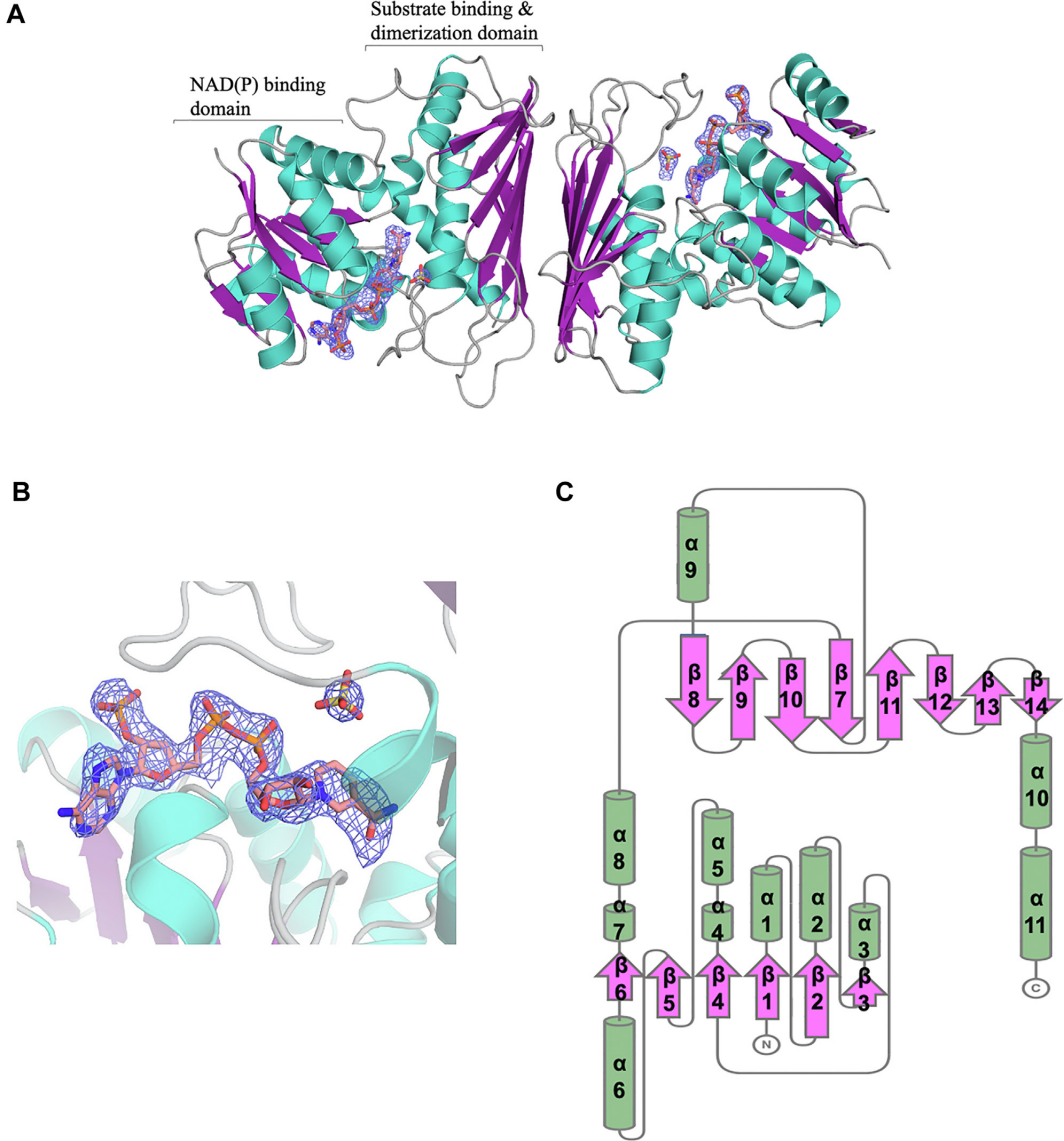
**Crystal structure of the PmdC dimer at 2.34 Å resolution.***A*, structural model of PmdC colored according to secondary structure: α-helices in *green*, β-strands in *purple*, and loops in *gray*. Each monomer bound to NADP reveals a two-domain architecture consisting of a conserved NAD(P)-binding domain and a substrate binding and dimerization domain. Bound ligands NADP and SO4 along with their simulated map density (*blue* mesh) are displayed for each monomer. Ligand omit maps were generated using polder maps from the Phenix computational crystallography suite. *B*, zoomed-in polder map representation of electron density around NADP (ChainC), the map is contoured to 2.0 σ. *C*, corresponding topological diagram for each monomer displaying the two-domain architecture. PmdC has a total of 11 α-helices; helices α1 - α8 are part of the N-terminal NAD(P)-binding domain, the remaining three helices α9-α11 are part of the substrate binding and oligomerization domain. Similarly, beta strands β1-β6 form a parallel β-sheet in the N-terminal domain, while strands β7-β14 form an antiparallel β-sheet that forms the dimerization interface. The topology map was generated using PDB sum and adapted using Topdraw in the CCP4 Molecular Graphics suite.

Purification of PmdC yielded high-purity protein that crystallized in both apo- and holo- (NADP bound) forms as clusters of thin plates (Fig. S4). BLASTP (25) analysis of the PmdC sequence revealed very low sequence similarity to proteins in the PDB (>30%), therefore an Alphafold (26) prediction of the protein structure followed by molecular replacement was used to solve the structure. The crystal structure of PmdC bound to NADP was solved to a resolution of 2.34 Å, spanning 98.75% of the protein sequence, Lys 3–Asn 317 (Fig. 3*A*). A close-up of the electron density around the NADP ligand is shown (Fig. 3*B*). Data collection, refinement, and model statistics for PmdC are summarized in Table 2. Each asymmetric unit contained six copies of PmdC, existing as three dimers. Size-exclusion chromatography confirmed the occurrence of PmdC as a dimer in solution (Fig. S5). The crystal structure revealed dimerization occurs at the interface between the C-terminal ß-sheets of each monomer. Each monomer has two domains: An N-terminal cofactor-binding domain bound to NADP (residues 1–141) and a C-terminal substrate binding and oligomerization domain (residues 145–317).

**Table 2:** Data collection and refinement statistics for PmdC-NADP (PDB code 9AZO)

Crystal parameters^a^	
Space group	P 1 2_1_ 1
Unit cell:	
a, b, c (Å)	79.151, 157.864, 95.011
α, β, ɣ (°)	90, 114.449, 90
Data collection statistics:	
Wavelength (Å)	1.00
Resolution range (Å)	29.44–2.342 (2.4–2.34)
R-meas	0.2728 (1.025)
Mean I/sigma(I)	2.90 (0.88)
CC1/2	0.948 (0.515)
Completeness (%)	97.66 (79.50)
Multiplicity	1.7 (1.7)
Refinement and model statistics:	
Reflections used in refinement	86,964 (5050)
R-work	0.2424 (0.3114)
R-free	0.2777 (0.3254)
Number of nonhydrogen atoms:	15,371
Macromolecules	14,688
Ligands	323
Solvent	360
RMS(bonds)	0.010
RMS(angles)	1.52
Ramachandran favored (%)	97.44
Ramachandran allowed (%)	2.50
Ramachandran outliers (%)	0.05
Rotamer outliers (%)	0.00
Clashscore	7.14
Average B-factor	44.14
Macromolecules	44.16
Ligands	45.86
Solvent	41.35
Ramachandran plot Z-score, RMSD:	
Whole (N = 1878)	0.37 (0.18)
Helix (N = 726)	0.46 (0.17)
Sheet (N = 456)	0.13 (0.22)
Loop (N = 696)	0.36 (0.25)
CaBLAM validation	
CaBLAM disfavored (%)	2.8
CaBLAM outlier (%)	0.2
CA geometry outlier (%)	0.48

a One crystal of PmdC soaked with NADP was used for the dataset.

#### The tertiary protein architecture in PmdC reveals structural similarity to the Gfo/Idh/MocA oxidoreductase protein family

By sequence analysis using Interpro (27), PmdC is classified under the Rossmann fold-NAD(P)^+^ binding oxidoreductase superfamily, sharing common structural characteristics with the Gfo/Idh/MocA oxidoreductase protein family (28). Though the sequence identity between PmdC and these proteins is low, their three-dimensional protein architecture is strikingly similar. Super-positioning PmdC with these protein structures yielded all-atom RMSDs ranging from 1.87 to 2.36 Å (Fig. S6). Key features shared between PmdC and the Gfo/Idh/MocA superfamily include a characteristic two domain architecture. As with other members of this family, the N-terminal of PmdC contains the NAD(P)^+^ nucleotide binding Rossmann fold. Another common feature is the organization of the C-terminal α/ß domain; where the first ß-strand of the ß-sheet (ß7) lies at the center of the sheet, parallel to one neighboring strand and antiparallel to the next (Fig. 3*C*). PmdC also has a conserved α-helix (α9) located immediately after the first ß-strand (ß7), which carries critical residues for catalysis. These common features indicate PmdC has structural characteristics common with the Gfo/Idh/MocA superfamily. In addition, substrates comprising cyclic or heterocyclic rings are commonly used substrates by proteins in this superfamily. The hemiacetal form of CHMS being heterocyclic fits into this group of substrates.

#### The N-terminal cofactor-binding domain reveals key interactions between PmdC and NADP

**Figure 4:**
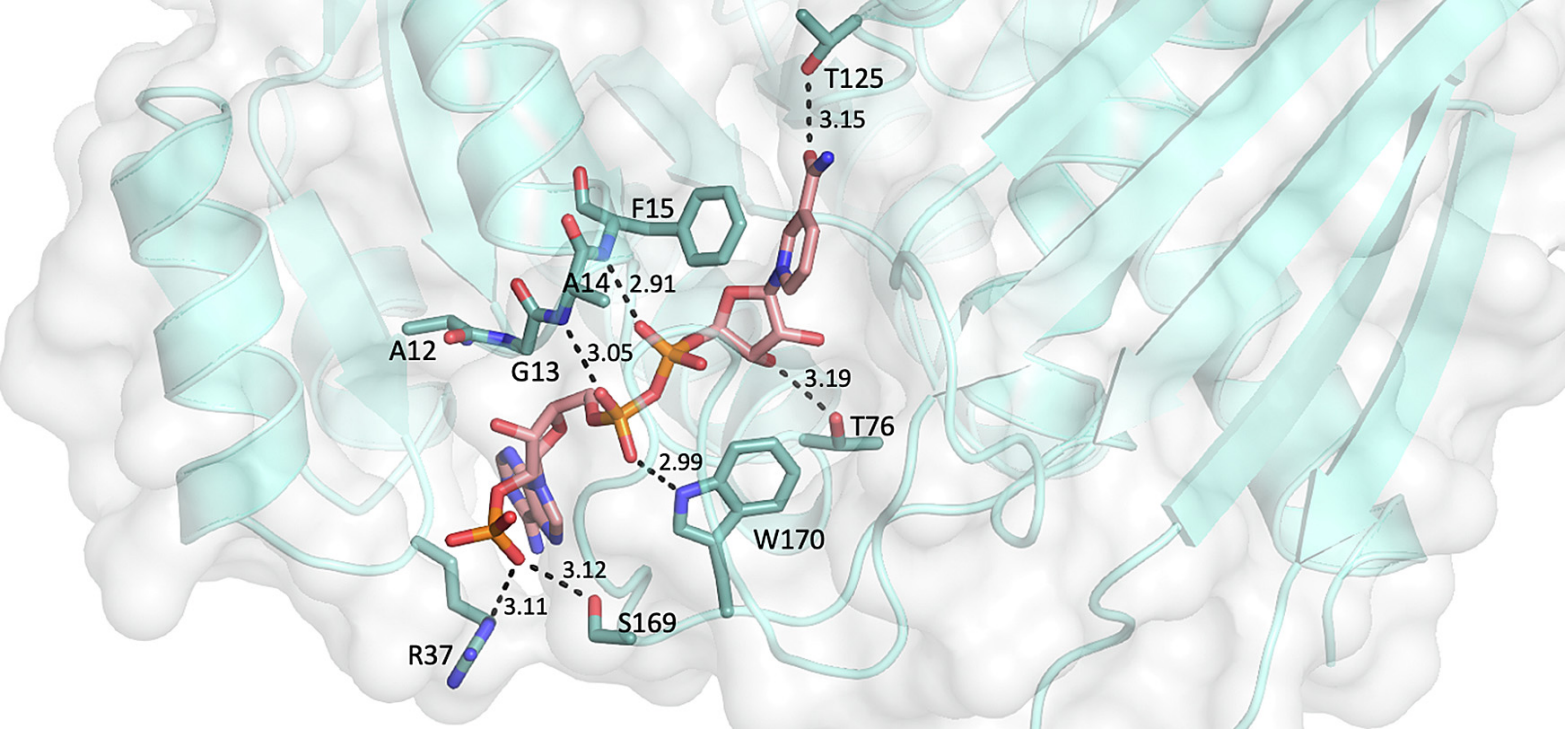
**Key binding interactions between PmdC and cofactor NADP.** The phosphate group of NADP is stabilized by Arg 37 and Ser 169, suggesting a preference for NADP over NAD at the molecular level. Interactions between the GXGXXG residues (Ala12 – Phe15) of the highly conserved β1−α1 loop in the N-terminal cofactor-binding domain and the diphosphate backbone of NADP are visible. The indole side chain of Trp 170 also participates in hydrogen bond interactions with the phosphate group Pa of the diphosphate moiety. The nicotinamide ring of NADP is involved in pi-stacking interactions with Phe 15 and hydrogen bond interactions with the hydroxyl side-chain of Thr 125 while the 3′-OH of the nicotinamide ribosyl group is stabilized by hydrogen bonding with the hydroxyl side chain of Thr 76.

The N-terminal NAD(P)-binding domain has a Rossmann fold consisting of alternating beta strands and alpha helices. The six ß-strands are unidirectional and are organized into a parallel ß-sheet flanked on either side by α-helices forming the well-known αßα sandwich motif (Fig. 3, *A* and *C*). PmdC contains the highly conserved ß1-α1 loop region at the start of the N-terminus which has the cofactor-binding glycine-rich consensus sequence GXGXXG (29, 30). Residues within this sequence participate in stabilization of the diphosphate backbone of NADP (31). This includes hydrogen bond and electrostatic interactions between the main-chain peptide backbone of Ala12, Gly13, and Ala14 with the diphosphate group of NADP (Fig. 4). In addition, hydrogen bond interactions are observed between the indole side chain of Trp170 with the phosphate group proximal to the adenosine moiety. Hydrogen bond interactions between PmdC and NADP involved in stabilizing the adenosine 2′-phosphate group are also observed. These interactions are mediated by the positively charged side chain group of Arg37 and the hydroxyl side chain of Ser169. This interaction likely contributes to a preference for NADP over NAD by PmdC. Interactions with the nicotinamide ring include pi-stacking interactions with the aromatic ring of Phe15 and hydrogen bond interactions between the hydroxyl side chain of Thr125 & the carboxyl group of the nicotinamide ring. Hydrogen bond interactions are also observed between Thr76 with the 3′-OH group of the nicotinamide ribosyl group. A Ligplot (32) representation of interactions between PmdC and NADP is shown in Fig. S7.

NADP is bound to PmdC within a tube-like cavity formed at the interface between the N-and C-terminal domains (Fig. S8). This cavity measures approximately 20 Å in height, 6.5 Å in width, 624 Å^3^ in volume, and has two openings at the ends of the tube. One opening is located close to the initial conserved ß1-α1 loop likely facilitating cofactor binding; the other opening is located at the middle of the molecule close to the nicotinamide ring possibly facilitating substrate binding/product diffusion. NADP is bound to PmdC along the length of the tube.

#### Molecular modeling of CHMS into the C-terminal domain reveals key residues involved in catalysis

A major part of the C-terminal domain consists of the dimerization interface comprising an antiparallel ß-sheet consisting of seven ß-strands. The three α-helices within this domain are located on the inner side of the ß-sheet creating a αß motif. The loop formed between the first ß−strand, ß7, and helix α9 of the C-terminal domain plays an essential role in substrate binding and contains key catalytic residues. This feature is also observed among other members of the Gfo/Idh/MocA protein family. Structural data revealed density within this region that was best fit with a sulfate ion from within the crystallization solution (Fig. 3).

**Figure 5:**
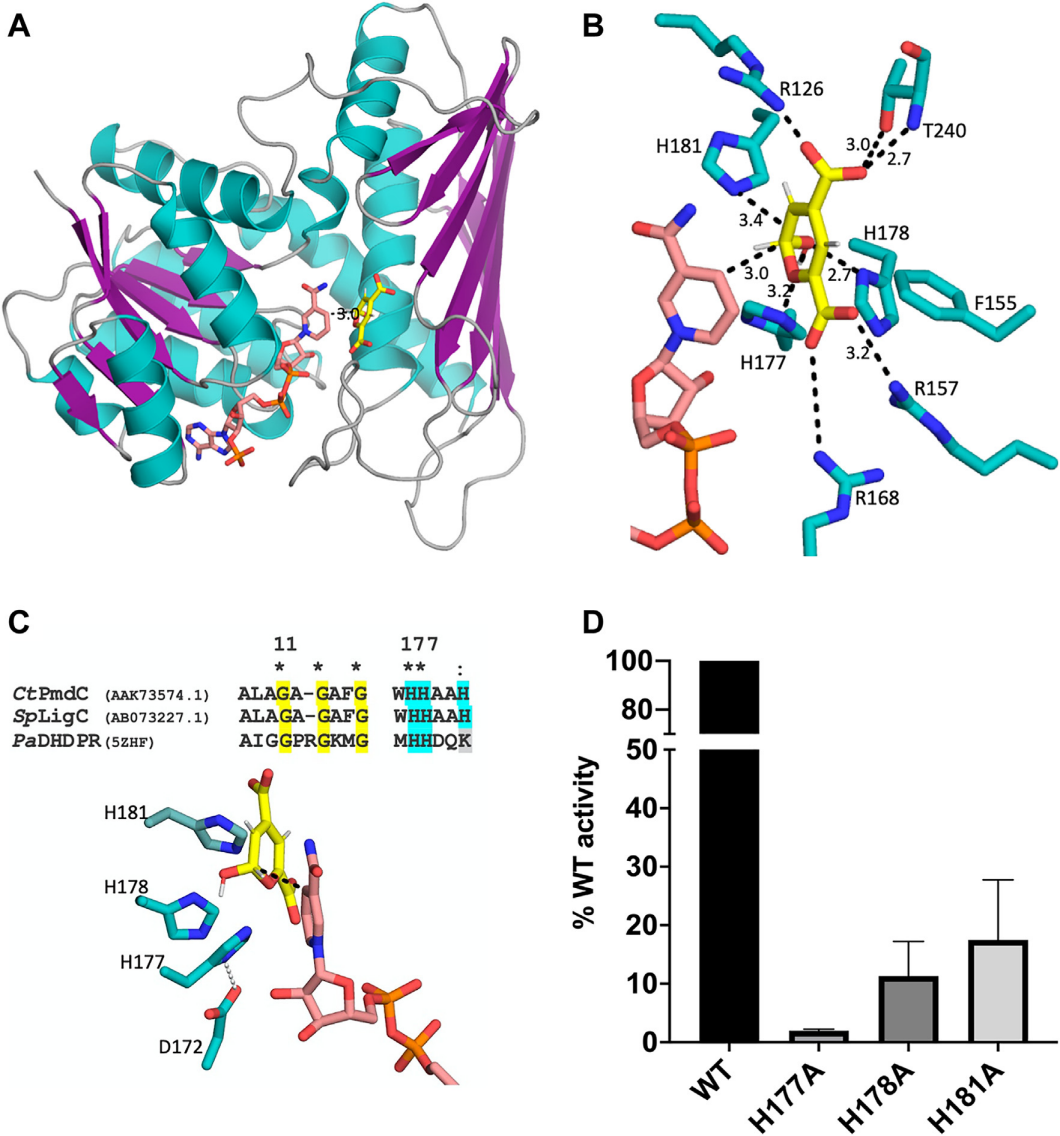
**CHMS modeled into the PmdC-NADP structure reveals key residues involved in substrate stabilization and catalysis.***A*, the C1 H-atom of CHMS (*yellow*) is oriented at a distance of 3.0 Å from C4 of the nicotinamide ring of NADP allowing hydride transfer. *B*, residues involved in the stabilization of CHMS at the active site, adapted from Ligplot analysis. All distances are in Å units. *C*, key residues likely involved in catalysis facilitating transfer of H-atom from C1 of CHMS to C4 of NADP, interactions between H177 and D172 are also shown. *D*, mutation of histidine residues to alanine results in a loss of activity in comparison to WT PmdC. Each bar represents the mean and SD from three independent experiments.

The CATH database (33) annotated the C-terminal domain of PmdC as belonging to the DHDPR domain superfamily. The three dimensional structures of DHDPR from *Paenisporosarcina* (PDB 5Z2F) (34) or *E*. *coli* (PDB 1ARZ) (23) are similar to PmdC in spite of a low approximately 20% sequence identity. In the crystal structures of the ternary complex of DHDPR containing both NADPH and ligand dipicolinate (DPA), the position of bound DPA is located in the same conserved region between the first ß-strand (ß7) and α-helix (α9) of the C-terminal domain. DPA is structurally analogous to CHMS, consisting of a nitrogen-containing heterocyclic ring and two carboxylic acid side groups at the C2 and C6 positions. CHMS in the hemiacetal form has an oxygen-containing heterocyclic ring and a hydroxyl group at the C2 position in addition to two carboxylic acid groups at C4 and C6 positions. Comparing the DPA position in DHDPR and the position of the electron density around the sulfate ion in PmdC, the hemiacetal form of CHMS was modeled into PmdC using Autodock. Two poses of CHMS were obtained with the lowest binding energies of −5.08 kcal/mol (pose1) and −5.05 kcal/mol (pose 2), (Fig. S9). Quantum Mechanical Restraints was subsequently used to optimize the two poses, of which pose 1 had a 40 kcal/mol lower energy than pose 2. For pose 1, both stereoisomers, that is, (R)- and (S)-2-hydroxy-2*H*-pyran-4,6-dicarboxylic acid, were also optimized. The (S)-isomer was associated with a higher binding energy of −233.836 kcal/mol, while the (R)-isomer was associated with a lower binding energy of −251.526 kcal/mol. However, there were clashes detected between the -OH group of the (R)-isomer with the nicotinamide ring of NADP (Fig. S10); therefore, (S)-isomer was used for subsequent analyses. Ligplot (32) analysis of the docked substrate and PyMol modeling revealed key residues involved in substrate binding and catalysis (Fig. 5).

In the model of CHMS bound to PmdC-NADP, C4 of the NADP nicotinamide ring is located at a 3.0 Å distance from the C2 carbon of CHMS in an orientation favorable for H-atom transfer (Fig. 5*A*). The carboxylic acid group attached to the C4 carbon in CHMS is bound by hydrogen bond interactions between the side chains of Arg 126 and Thr 240 and the main chain peptide N-atom of Thr 240, while the second carboxylic acid group attached to C6 is bound by interactions with the side chains of Arg 157 and Arg 168. Hydrophobic interactions with Phe 155 appear to be involved in stabilization of the CHMS ring (Fig. 5*B*). The hydroxyl group attached to C2 carbon interacts with the side chain groups of His 177, His 178, and likely His 181 at hydrogen bond distances. While all three histidine residues are conserved between PmdC and *Sp*LigC SYK-6, only two are conserved in DHDPR enzymes, the third residue being a lysine (Fig. 5*C*). In *Ec*DHDPR, the consensus sequence ^157^E(L/A)HHXXKXDAPSGTA^171^ is proposed to represent the substrate-binding site. The two histidine and lysine residues in the sequence are conserved among the DHDPR enzymes and are reported to play an essential role in catalysis. In *Ec*DHDPR, His159 is proposed to act as a general acid, involved in proton transfer to the substrate *via* an activated water molecule, His160 is involved in direct interactions with the C2 carboxylate group and Lys163 interacts with the ring imine nitrogen, polarizing the substrate for hydride transfer and stabilizing the intermediate enamine during substrate reduction. Mutating the His159 and Lys163 residues led to a dramatic drop in activity indicating a role in both substrate binding and catalysis (23).

Like DHDPR, PmdC also has a stretch of residues ^168^RSWTDHLLWHHXXHXXDXF^186^ which are conserved across homologs and contains residues involved in substrate binding. A sequence alignment of PmdC with *Sp*LigC and the 16 CHMS dehydrogenases tested for PDC production by Zhou *et al*. (18) reveals highly conserved stretches of residues in the CHMS dehydrogenases corresponding to the substrate binding site (Fig. S11). The structure of PmdC-NADP with docked CHMS reveals that the three histidine residues in the conserved substrate-binding sequence play an essential role in catalysis. Mutation of each histidine residue to alanine resulted in a substantial drop in activity. The His177A mutant exhibited a 98% loss of activity while the H178A and H181A mutants showed an 89% and 83% loss of activity respectively compared to WT PmdC (Fig. 5*D*). These results suggest a catalytic role for the H177 residue while the H178 and H181 residues are likely involved in substrate stabilization during hydride transfer to NADP^+^. Using size-exclusion chromatography, each of the three mutant proteins eluted at a volume similar to that of WT PmdC, indicating no significant changes in three-dimensional protein structure resulting from swapping histidine residues with alanine (Fig. S12).

#### Active site histidine residues drive NAD(P) reduction likely *via* acid base catalysis

**Figure 6:**
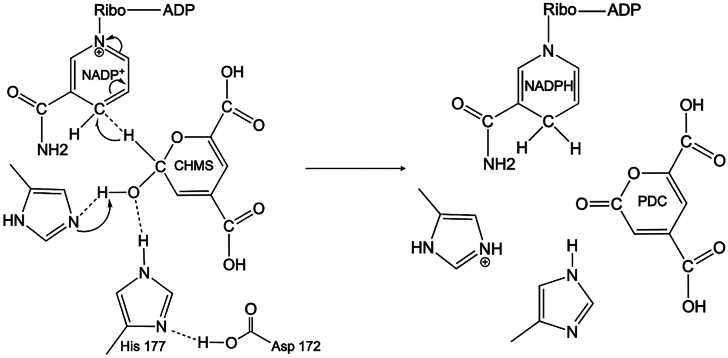
**Proposed mechanism for the PmdC catalytic cycle.***Left panel*, in the first part of the reaction, NADP^+^ abstracts one H-atom from the CHMS substrate aided by neighboring histidine residues: H177 is likely the catalytic acid while the other two histidine residues likely participate in substrate stabilization during catalysis. Interactions between H177 and D172 are also shown. *Right panel*, these interactions in concert catalyze the production of PDC with the generation of NADPH.

The three histidine residues H177, H178, and H181 are in close proximity to the hydroxyl group & hydrogen atom of the C2 carbon atom of CHMS involved in NADP^+^ reduction. QM calculations were used to determine the optimum rotamer conformation for each of the three histidine residues in its neutral protonated state Nε2 alone, Nδ1 alone, or biprotonated Nε2 Nδ1state (Table S4). For all three histidine residues, the lowest energy conformation consisted of a singly protonated nitrogen atom, consistent with the slightly basic buffer of pH 7.5. In the optimized conformation of His177, the H-atom bound to Nε2 is within hydrogen bonding distance to both the hemiacetal ring oxygen atom and the C2 hydroxyl group while the Nδ1 atom is involved in electrostatic interactions with Asp172, enhancing its acid properties (Figs. 5*C* and S13). Site-directed mutagenesis of the H177 residue to alanine results in an almost complete loss of activity indicating that the residue likely plays a role as a catalytic acid. In the DHDPR enzymes, a conserved Glu residue E157 *Ec*DHDPR (23) or E154 PaDGDPR (34) is involved in similar electrostatic interactions with the catalytic histidine residue. In the optimized conformation of His178, Nδ1 is protonated, while in H178, Nε2 is protonated. Both residues are within 3.0 to 3.4 Å to the C2 hydroxyl group of CHMS. Mutation of these residues to alanine resulted in significantly decreased activity, with only 11% activity retained for H178 and 17% activity retained for H181 residue respectively, compared to WT. These results suggest residues H178 and H181 likely play a role in substrate stabilization during catalysis. While substrate docking and QM calculations provided invaluable insight into residues involved in substrate binding and catalysis, the sequence of events involving hydride transfer to NADP^+^, subsequent stabilization of the product molecule, involvement of a catalytic base and possible participation of water molecules during catalysis remain to be determined. Distances between H177, H178, and H181 in the optimized conformations are shown in Fig. S13. A schematic representation of the proposed mechanism is shown in Figure 6.

## Discussion

The protocatechuate 4, 5 cleavage pathway is the main pathway for the bioproduction of PDC occurring *via* the oxidation of CHMS by enzyme CHMS dehydrogenase. In this study, we presented the first crystal structure of a CHMS dehydrogenase bound to cofactor NADP. While the enzyme has a reported higher affinity for NADP with Km that is 10 to 15 times lower than that for NAD, it is capable of using both NAD or NADP cofactors at nonlimiting concentrations. The structure reveals two residues Arg 37 and Ser 169 involved in binding the 2′-phosphate group of NADP likely contributing to a greater affinity for NADP. The presence of charged residues that bind the 2′-phosphate group is common among enzymes with a higher specificity for NADP. Enzymes with specificity towards NAD usually have an acidic residue such as aspartate that binds the 2′ and 3′ hydroxyl groups of the ribosyl moiety of adenosine, creating a repulsion with the 2′-phosphate of NADP (35). This residue is absent in PmdC; however, interactions common to both NADP and NAD include main chain interactions between the protein backbone of the GXGXXG consensus sequence, together with side chain residue interactions stabilizing the diphosphate backbone and nicotinamide ring. NADP is metabolically more expensive than NAD, and successful swapping of cofactor specificity between NAD and NADP has been previously reported in other NAD(P)-binding proteins (36). The structure of NADP-bound PmdC could aid protein engineering efforts to change cofactor specificity for PDC production, for improved yields.

The binding site of CHMS was determined by a combination of structural and computational methods. Modeled CHMS is oriented within the PmdC active site such that the C2 atom bound to the hydroxyl group is oriented at a distance of 3.0 Å from the C4 atom of the nicotinamide moiety facilitating H-atom transfer. The C2 hydroxyl group of CHMS is also within hydrogen bonding distance of three key histidine residues H177, H178, and H181. Site-directed mutagenesis of these residues to alanine shows a near complete loss of activity with the H177A mutant, while the H178A and H181A mutants retained less than 20% activity. Based on these results, we hypothesize that H177 acts as a catalytic acid, while the two remaining histidine residues possibly play a role in substrate stabilization during catalysis. Sequence alignments of CHMS dehydrogenases revealed a highly conserved substrate-binding region containing a core ^177^HHXXH^181^ sequence (PmdC numbering), which are conserved across CHMS dehydrogenases. A similar ^159^HHXXK^163^ sequence (*Ec*DHDPR numbering) is found at the core of a conserved substrate-binding sequence in DHDPR proteins (23, 34). As with PmdC, the two histidine and lysine residues in DHDPR have been shown to be involved in catalysis. The structure of DHDPR was solved in the presence of bound NADP and inhibitor DPA in *E*. *coli* and *Paenisporosarcina* TG-14 homologs (23, 34). Both DPA and CHMS have a similar structure, and the two proteins have a similar three-dimensional architecture despite having low sequence identity. The holo crystal structure of *Ec*DHDPR yielded a detailed mechanism of catalysis showing that H159 acted as a catalytic acid for providing protons to the reduced substrate *via* an activated water molecule while K163 stabilized the ring nitrogen atom during catalysis (23). Our crystal structure with CHMS docked at the active site yielded important insight into residues involved in substrate binding and catalysis; however, mechanistic details with respect to stabilization of the substrate by H178 and H181 residues, participation of a catalytic base, and the possible involvement of water molecules during catalysis are to be elucidated. Owing to the inherent instability of the CHMS molecule, obtaining a crystal structure of the ternary complex with bound NADP and CHMS is a challenge, and alternative substrate analogs will be explored. This work will inform further investigation into the catalytic mechanism of PmdC.

In conclusion, our study adds to the repertoire of characterized microbial ligninolytic enzymes within pathways that breakdown lignin aromatics into products of value. The structural and biochemical characterization of PmdC will aid in the optimization of metabolic pathways aimed at producing PDC at high yields, thereby facilitating the use of lignin-derived biomass towards the sustainable production of high-value products which is crucial towards achieving a renewable bio-based economy.

## Experimental procedures

Unless otherwise mentioned, chemicals in this study were of the highest purity available and were used as received.

### Cloning, expression, and purification of PmdAB and PmdC

Bacterial strains and plasmids are listed in Table S1. Strains and plasmids along with their associated information (annotated GenBank-format sequence files) have been deposited in the JBEI Registry (https://registry.jbei.org). Full-length *pmdC* from *C*. *testosteroni* (GenBank AAK73574.1) was optimized for expression in *E*.*coli* using the Genscript codon optimization tool, synthesized by Integrated DNA Technologies (IDT), and cloned into a modified pSKB3 vector between *Nco*I and *Bam*HI restriction sites. Oligonucleotide primers for plasmid sequencing (Table S2) were synthesized by IDT. Restriction enzymes were purchased from Thermo Fisher Scientific, Phusion DNA Polymerase & Gibson assembly master mix were purchased from New England Biolabs, and were used for DNA amplification and to ligate full-length *pmdC* into the digested pSKB3 backbone respectively. The resulting construct encoded an N-terminal His6-PmdC protein with a Tobacco Etch Virus (TEV) protease recognition sequence inserted between the His6-tag and *pmdC* for tag removal. Similarly, full-length *pmdAB* from the same species (GenBank: AF459635.1) was codon optimized and cloned resulting in a construct encoding an N-terminal His6-PmdA and PmdB protein. The presence of a consensus RBS sequence (AGGAAG) before the stop codon of *pmdA* ensured co-expression of PmdA and PmdB from the same plasmid. Constructs were transformed into chemically competent *E*. *coli* BL21(DE3) strains (New England Biolabs) and grown on Lysogeny Broth (LB) agar plates under 50 μg/ml Kanamycin selection (LB Kan-50 plates). Transformants were grown in LB broth under Kanamycin (Teknova) selection, plasmid extractions were carried out with Qiagen miniprep kits, and sequence verified *via* Genewiz, Azenta Life Sciences. Transformant cultures were stored as 1 ml glycerol stocks at −80 °C.

For overexpression of PmdC, a frozen glycerol stock was used to inoculate 50 ml of LB broth containing 50 μg/ml Kanamycin. The starter culture was incubated overnight at 37 °C with constant shaking at 200 rpm. For large scale growth, the starter culture was diluted 100-fold in a 2-L baffled shake flask containing 1 L of LB broth supplemented with 50 μg/ml Kanamycin and grown at 37 °C with constant shaking at 190 rpm. At OD600 nm ∼0.7, the culture was induced with IPTG to a final concentration of 0.5 mM. Approximately 30 min after induction, the temperature was dropped to 18 °C and cells were allowed to grow overnight for ∼18 h. Cells containing overexpressed protein were harvested by centrifugation and cell pellets were stored at −80 °C until lysis. PmdAB was overexpressed in the same manner.

For purification, PmdC cell pellets were resuspended in lysis buffer containing 25 mM Hepes (pH 7.5, Sigma), 250 mM NaCl (VWR BDH Chemicals), 10 mM imidazole (MilliporeSigma), and 1 mM DTT (VWR) supplemented with chicken-egg lysozyme (300 ug/ml, MilliporeSigma), DNaseI (50 ug/ml, MilliporeSigma), and powdered protease inhibitor tablets (Pierce EDTA-free tablets, Thermo Fisher Scientific). This mixture was incubated for 30 min followed by sonication (Qsonica; Thomas Scientific) on ice, inside a cold room. Pulses were set at 35% amplitude with repeated cycles comprising a pulse-on time of 15 s followed by a pulse off time of 45 s for a total lysis time of 10 min. Lysates were then clarified by centrifugation (Beckman-Coulter) at 18,000 rpm at 4 °C. Clarified lysates were filtered through a 0.45 *μ*m syringe filter (Nalgene, Thermo Fisher Scientific) and loaded on a 5 ml His Trap HP column (Cytiva) pre equilibrated with buffer A comprising 25 mM Hepes (pH 7.5), 250 mM NaCl, 20 mM imidazole, and 1 mM DTT on an FPLC system (AKTA explorer, Cytiva). The column was then washed with five Column Volumes (CV) of buffer A to remove unbound proteins and eluted using a linear gradient formed by mixing buffer A with buffer B which contained 25 mM Hepes (pH 7.5), 250 mM NaCl, 500 mM imidazole, and 1 mM DTT. PmdC eluted at a concentration of 25% B, the presence of PmdC in elution fractions were confirmed by SDS-PAGE (BioRad) and proteomics on SDS-PAGE gel slices (37). Fractions with minimal contaminants were pooled and buffer exchanged using a PD-10 column (Cytiva) pre-equilibrated with buffer C containing 25 mM Hepes (pH 7.5), 150 mM NaCl, 1 mM DTT. The protein was then incubated overnight with in-house purified TEV protease at a concentration of 1:10 w/w protein/TEV protease at 4 °C. Protein cleavage was verified by SDS-PAGE. The protein, TEV protease mix was then concentrated to 1 ml using a 10 kDa cutoff Amicon protein concentrator (Millipore Sigma) and diluted to a volume of 50 ml in buffer D containing 25 mM Hepes (pH 7.5), 10 mM NaCl, 1 mM DTT, then filtered through a 0.45 *μ*m syringe filter, and loaded onto a 5 ml HiTrap Q HP column (Cytiva) pre-equilibrated with buffer D. The column was washed with five CV of buffer D and eluted using a linear gradient formed by mixing buffer D with buffer E which contained 25 mM Hepes (pH 7.5), 1M NaCl, and 1 mM DTT. PmdC eluted at a concentration of 12.5%B while TEV protease did not bind the column and eluted in the flow through. As a final purification step, fractions that showed minimal contaminants by SDS-PAGE were pooled, concentrated to 0.5 ml, and loaded on a 120 ml Superdex 200 HiLoad 16/60 size-exclusion column (Cytiva) pre-equilibrated with buffer C. PmdC was eluted isocratically in buffer C. Purity of fractions was assessed by SDS-PAGE, and protein concentration was determined using Bradford Assays. Proteins were used immediately for crystallization and activity assays. Leftover protein was aliquoted & flash frozen in liquid nitrogen and stored at −80 °C until use.

Purification of PmdAB was performed within an anaerobic chamber (Coy Laboratory Products) filled with a gas composition of 85% N_2_/10% CO_2_/5% H_2_ (ultra-high purity, anaerobic mixture) and maintained at ambient temperature (∼22 °C). Glass, plastic, and stainless-steel materials used for purification were allowed to degas in the glove box for at least 1 day before use, as were heat labile solids. All buffers and solutions were prepared in 18-MΩ resistance double distilled water (Barnstead Nanopure system, Thermo Fisher Scientific), which was autoclaved and purged with ultra-high purity N_2_ gas and stored within the anaerobic chamber. For purification, 1L culture pellet was resuspended in lysis buffer containing 25 mM Hepes (pH 7.5), 250 mM NaCl, 10 mM imidazole, and 1 mM DTT supplemented with chicken-egg lysozyme (300 ug/ml), DNaseI (50 ug/ml), and powdered protease inhibitor tablets. The cells were incubated for ∼30 min in buffer and then lysed using a 30 ml Potter-Elvehjem homogenizer (Thermo Fisher Scientific). Lysates were clarified by centrifugation at 18,000 rpm for 20 min, filtered through a 0.45 *μ*m syringe filter, and loaded onto a benchtop 1.5 × 10 cm Econo-column (Bio-rad) filled with 1 ml Ni-NTA agarose beads (Qiagen) pre-equilibrated with buffer A within the anaerobic chamber. Unbound proteins were washed out with five CV of buffer A and an increasing stepwise imidazole concentration of 50 mM and 75 mM was prepared by mixing buffers A and B at five CV for each imidazole concentration. After the washing steps, PmdAB was eluted in five CV of buffer B. The presence of PmdAB in elution fractions was confirmed by SDS-PAGE. Fractions were pooled and buffer exchanged using a PD-10 column into buffer C. Protein concentration was determined by the Bradford assay, the protein was then aliquoted, flash frozen, and stored at −80 °C until use. The identity of both proteins was confirmed using proteomics on SDS-PAGE gel slices of purified proteins (37).

Site-directed mutagenesis was used to create PmdC mutants H177A, H178A, and H181A. The QuikChange Lightning kit (Agilent) was used with protocols as recommended by the manufacturer. Plasmid pSKB3-pmdC was used as a template; primers containing the appropriate mutations were ordered from IDT (Table S2). Mutations incorporated into the *pmdC* gene were confirmed by DNA sequencing (Genewiz, Azenta Life Sciences), and the resulting plasmids were individually transformed into chemically competent *E*.*coli* BL21DE3 cells (New England Biolabs). Growth and protein purification protocols for the mutant PmdC proteins are identical to WT PmdC.

### PmdC activity using UV-Visible enzymatic assays

The activity of PmdC was determined by UV-Visible spectrophotometry assays using 96-well UV-transparent plates (Sigma Aldrich); plates were read using a Spectramax M2 plate-reader (Molecular Devices). Initial experiments for path length confirmation of plate wells were performed using 0.1 mM NADH (ß-nicotinamide adenine dinucleotide, reduced disodium salt, Millipore Sigma) in a volume of 200 *μ*l water at 340 nm and a molar extinction coefficient of 6220 M^−1^ cm^−1^. To determine the molar extinction coefficient of CHMS, stocks of 0.05 to 0.15 mM PCA (3,4 dihydroxybenzoic acid, MP Biomedicals) dissolved in 10 mM phosphate buffer (pH 7.5) was mixed with 10 *μ*M PmdAB in buffer C to a volume of 190 *μ*l. The rise in absorbance from CHMS accumulation was recorded at 410 nm until the signal leveled off in approximately 10 min. At this point, an endpoint reading was recorded which was followed by the addition of 10 *μ*l 3N NaOH. An endpoint reading was once again recorded of the increase in absorbance upon NaOH addition. Using the previously reported molar extinction coefficient of CHMS in NaOH of 29,000 M^−1^ cm^−1^ (24), the molar extinction coefficient of CHMS in buffer was determined to be 2547 M^−1^ cm^−1^. A day prior to running assays, flash frozen PmdAB was thawed in the anaerobic chamber, concentrated to 250 *μ*M using a 10 kDa molecular weight cutoff Amicon concentrator, incubated with 0.5 mM Dithionite, and placed within a sealed Desi-Vac container (Thermo Fisher Scientific) at 4 °C until use. For the enzymatic assays, PCA stock concentrations of 0.033, 0.055, 0.11, 0.22, 0.33, 0.44, and 0.55 mM in 10 mM phosphate buffer (pH 7.5) were prepared. At higher PCA concentrations, protein precipitation was observed; hence, 0.55 mM was chosen as the maximal concentration. To each well of the 96-well plate, 180 *μ*l of each PCA stock was added in triplicate along with a buffer control with no PCA. PmdAB was added to a concentration of 12.5 *μ*M and kinetic spectra of the rise in absorbance at 410 nm was recorded over 40 min. After 40 min, an endpoint reading was recorded. Immediately after, 1.3 *μ*M PmdC pre-incubated with 0.81 mM NADP (ß - nicotinamide adenine dinucleotide phosphate hydrate, Sigma) or NAD, (ß - nicotinamide adenine dinucleotide, Sigma Aldrich) were added to the reaction mix and kinetic spectra of the decrease in absorbance at 410 nm was recorded over 40 min, followed by an endpoint reading. Controls with no PmdC or no cofactor were also run. Data were collected on SoftMax Pro software v7.0.3. For kinetics experiments, data were collected at room temperature; data points were recorded every 20 s at 410 nm for a total of 40 min, with shaking every 3 s. Three independent experiments were performed and data were analyzed and plotted using MS Excel v16.39 and Graphpad Prism v9.5.0.

### LC-MS–based PmdC enzymatic assays

All solutions were prepared in LC-MS-grade water (Honeywell) or methanol (Sigma-Aldrich). These assays were carried out as follows: PCA (3,4 Dihydroxybenzoic acid) was dissolved in water to a stock concentration of 20 mM and was later diluted to a working concentration of 10 mM in buffer C containing 25 mM Hepes (pH 7.5), 150 mM NaCl, 1 mM DTT for the assay. NADP was dissolved in LC-MS water to a final concentration of 27 mM. Both PmdAB and PmdC were dialyzed into buffer C for assays. Approximately 24 h prior to running the assay, 0.5 mM Dithionite was added to PmdAB, within the glove box and stored in a vacuum sealed container at 4 °C. Initial assays contained 0.05 mM PmdAB, 2 mM PCA, 0.05 μM PmdC, and 2 mM NADP in a volume of 0.6 ml. The mixture was placed on a tabletop shaker set at 30 °C and 400 rpm with constant shaking. Reactions were initiated by the addition of PmdAB or PmdC. Immediately upon initiation of the reaction and after every 20 min over a period of 100 min, 75 μl of reaction mixture was removed and heated to 100 °C for 10 min to inactivate the enzymes. This was followed by the addition of 75 μl 100% methanol. Samples were then centrifuged to 13,000 rpm for 15 min and then diluted 4-fold in 50/50 water/methanol. Each reaction was run in parallel with a replicate. For reactions measuring PDC, the reaction with PCA and PmdAB was allowed to progress for ∼15 min prior to the addition of PmdC.

For quantification of PCA consumed and PDC produced, six-point calibration curve standards of PCA and PDC were prepared in 50/50 water/methanol (v/v) at concentrations of 1.5, 3.0, 6.25, 12.5, 25, and 50 μM. PDC was a gift received from the laboratory of Professor Frank Raushel at Texas A&M University and was synthesized by Dr Tamari Narindoshvili (11). The PDC powder was dissolved in LC-MS grade water to a concentration of 13 mM and diluted to the concentrations of the aforementioned calibration standards. Samples were run as described previously (38) on an Agilent Technologies 1260 HPLC system coupled to an Agilent Technologies 6520 Quadrupole Time-of-Flight Mass Spectrometer. The expected monoisotopic *m/z* (negative ionization) of deprotonated PCA, CHMS, and PDC are 153.019332, 185.00916, and 182.993511, respectively. Peak areas were integrated using Agilent Technologies Mass Hunter Qualitative Analysis software (version B.06.00). Using this method, CHMS eluted as a mixture of ∼ 4 peaks. In order to convert CHMS into a uniform state, samples were re-run as described above, but in buffer acidified with 0.1% formic acid. Metabolite separation was performed using a Kinetex XB-C18 column (Phenomenex) of length 3 × 100 mm and 1.7 μm particle size. Column compartment and autosampler temperatures were set to 40 °C and 4 °C, respectively. The mobile phase used was 10 mM ammonium acetate with 0.1% formic acid in water (A) and 10 mM ammonium acetate with 0.1% formic acid in 90% acetonitrile, 10% water (B). A flow rate of 0.42 ml/min was used (unless stated otherwise) with gradient elution using the following mobile phase compositions: 5%B (at 0 min), 97.1%B (at 6.5 min), 97.1%B (at 7.8 min), 5%B (at 8.2 min), 5%B (at 10.2 min). The flow rate was linearly increased from 0.42 ml/min (at 7.8 min) to 0.65 ml/min (at 8.2 min) and held at this flow rate for an additional 2 min. The sample injection volume was set to 3 *μ*l. Electrospray ionization conditions were as follows: negative ion mode, drying gas temperature of 340 °C, drying gas flow rate of 11 L/min, nebulizer pressure of 30 psig, capillary voltage of 3500 V, fragmentor voltage of 100 V, skimmer voltage of 60 V, OCT 1 RF voltage of 200 V, and a data acquisition range of 50 to 700 m/z. For LC-MS/MS analysis of CHMS, a precursor ion of 185.00916 *m/z* was selected (at an isolation width of 1.3 *m/z*) for collision-induced dissociation at a collision energy of 20 eV. Acquisition rates for LC-MS and LC-MS/MS experiments were 0.86 spectra/s and 1 spectra/s, respectively.

### Crystallization of PmdC and structure solution

For crystallization, PmdC was concentrated to 40 mg/ml in a buffer containing 25 mM Hepes (pH 7.5), 150 mM NaCl, and 1 mM DTT. Trials were set up at concentrations of 40 mg/ml and 20 mg/ml in the apo- and holo-NADP bound forms. A 3-fold molar excess of ß - nicotinamide adenine dinucleotide phosphate sodium salt (Sigma Aldrich) was incubated with PmdC for 30 min prior to co-crystallization setup. Crystallization trials were performed using 96-format high throughput screens which included the following: Index, PEG/Ion, PEGRx, Crystal screen Cryo (Hampton Research), SG1 screen (Molecular Dimensions), MCSG1 screen (Anatrace), and Berkeley screen (Rigaku) at room temperature using the sitting drop format in 96 × 3 well Intelli-Plates (Art Robbins) at a 1:1 protein:crystallization solution ratio using a Phoenix robot (Art Robbins Instruments). Crystals were observed in several different conditions; these crystals were cryoprotected in a solution of 30% glycerol in mother liquor, then flash frozen in liquid nitrogen. X-ray diffraction data was collected both at the National Synchrotron Lightsource on the FMX beamline (Brookhaven National Laboratory) and the Berkeley Center for Structural Biology on beamline 8.2.2 of the Advanced Light Source (Lawrence Berkeley National Laboratory). Data on a total of 32 crystals were collected and the highest resolution dataset was obtained from a crystal grown in 0.2 M ammonium sulfate 0.1 M Bis–Tris pH 5.5, 25% w/v PEG 3350, condition F6 in the Index screen. Data processing was performed automatically at the FMX beamline using fastDP. Programs within the *Phenix* suite were used for solving the structure (39, 40). *Phenix*.*xtriage* (41) was used to check for anomalies in the processed data and determine the number of protein molecules in the asymmetric unit. Molecular replacement using *PHASER* (42) was used to determine the structure using a model of the protein derived from Alphafold (26) as the search model. NADP and SO_4_^2-^ were built into the structure using *Ligand*.*fit* (43) and *COOT* (44). Omit maps for ligands were made using *Polder*.*maps* (45). Structure refinement was performed using *Phenix*.*refine* (46); structure validation was performed using *Molprobity* (47). Manual correction of residue deviation from ideal stereochemistry was performed using *COOT*. During refinement, the Alphafold model of PmdC was used as a reference model (48); secondary structure restraints and torsion-angle noncrystallographic symmetry (49) were also used. In the final stages of refinement, TLS refinement was used, and X-ray/stereochemistry weights were optimized. Refinement statistics for PmdC are given in (Table 1). Structure images were generated using PyMol molecular graphics v2.2.3 (50) and CCP4MG v2.10.11 (51). Crystallization trials of PmdC crystals soaked in product PDC were attempted but were unsuccessful.

### *In silico* docking of CHMS to PmdC

The *Autodock Tools* suite (52) was used for *in silico* docking of CHMS to PmdC. A single chain (Chain C) of monomeric NADP-bound PmdC with overall complete electron density and with the sulfate ion and crystallographic waters removed was used as the macromolecule for docking. The three-dimensional structure of CHMS was obtained from Chemspider (http://www.chemspider.com) and was used as the ligand. Using *AutoDock Tools*, polar hydrogen atoms were added to PmdC, followed by Kollman charges. Polar hydrogen atoms were also added to the CHMS molecule followed by the addition of Gasteiger charges. Three torsional degrees of freedom were identified for CHMS. Following macromolecule and ligand preparation, the individual files were saved in the PDBQT format. A grid parameter file was generated by selecting a grid of 56, 40, and 60 points in the x, y, and z directions with a grid spacing of 0.375 Å. The grid was centered at the interface between the NADP-binding domain and the ligand-binding domains of PmdC (x = 12.941, y = −64.898, z = −30.706), based on ligand binding in the crystal structure of DHDPR (*Pa*DHPR, PDB 5Z2F), which shows structural and ligand similarity to PmdC. To generate the docking parameter file, the Lamarckian genetic algorithm was used for sampling ligand-binding conformations; docking parameters were set to default values. *Autogrid4* was used to generate affinity maps and *Autodock4* was used to perform docking simulations between macromolecule and ligand. A total of 100 docking simulations were performed. As a control, docking simulations of DPA to NADP-bound DHPR were also performed. The simulation with the lowest binding energy and favorable orientation was selected. Docking simulations were analyzed using *Autodock Tools* and were saved as PDB files. Docking was visualized using molecular modeling software BIOVIA Dassault Systèmes Discovery Studio (53) and PyMol v2.2.3 (50). Clashes between docked substrates were visualized using UCSF Chimera (54) at a Van der Waals overlap of 0.6 to 0.8 Å.

### Quantum chemistry

Using a model of the binding pocket to reduce the computational expense in a similar fashion to the Quantum Mechanical Restraints implementation (55) in the Quantum Interface module of Phenix, new code was written to determine the “best” protonation and orientation of the three histidine residues neighboring the ligand and both isomeric forms. The algorithm inserted the six protonation and orientations of each histidine in turn and minimized the geometry of the side chain atoms of the specific histidine along with all hydrogen atoms in the model. The QM method chosen was PM6-D3H4 (56) in the MOPAC (57) package that has been shown to perform well on organic molecules. The relative energies were used to determine the likely protonation state of each histidine.

## Data availability

Data deposition: The atomic coordinates and structure factors of PmdC bound to NADP have been deposited in the Protein Data Bank, www.pdb.org under code 9AZO.

## Supporting information

Supporting information is available as a separate file containing supporting figures and tables. This article contains supporting information (58).

## Conflict of interest

The authors declare that they have no conflicts of interest with the contents of this article.
