# Supplementary material for: Characterization of lignin-degrading enzyme PmdC, which catalyzes a key step in the synthesis of polymer precursor 2-pyrone-4,6-dicarboxylic acid
Source: J Biol Chem. 2024 Aug 31;300(10):107736. doi: 10.1016/j.jbc.2024.107736 (PMC11489326; doi:10.1016/j.jbc.2024.107736)
Supplement: Supplemental information [file mmc1.docx]

**Characterization of lignin degrading enzyme PmdC, which catalyzes a key step in the synthesis of polymer precursor 2-pyrone-4,6-dicarboxylic acid (PDC)**

# Supporting Information

Andria V. Rodrigues^1,2,*^, Nigel W. Moriarty^2^, Ramu Kakumanu^1,3^, Andy DeGiovanni^1,2^, Jose Henrique Pereira^1,2^, Jennifer W. Gin^1,3,4^,Yan Chen^1,3,4^, Edward E. K. Baidoo^1,3^, Christopher J. Petzold^1,3,4^ and Paul D. Adams^1,2,5,*^

^1^ Joint BioEnergy Institute, Emeryville, California, 94608, United States

^2^ Molecular Biophysics and Integrated Bioimaging, Lawrence Berkeley National Laboratory, Berkeley California 94720, United States

^3^ Biological Systems and Engineering Division, Lawrence Berkeley National Laboratory, Berkeley California 94720, United States

^4^ Department of Energy Agile BioFoundry, Emeryville, California United States

^5^ Department of Bioengineering, University of California Berkeley, Berkeley California 94720, United States

^*^ For correspondence: [pdadams@lbl.gov](mailto:pdadams@lbl.gov), [avrodrigues@lbl.gov](mailto:avrodrigues@lbl.gov)

**Table of Contents**

[*Supporting Information 1*](#_gjdgxs)

[Supporting figure 1: UV-Visible spectrum of PmdAB catalyzed conversion of PCA to CHMS. 3](#_30j0zll)

[Supporting figure 2: UV-Visible assays measuring the kinetics of PmdAB and PmdC. 4](#_1fob9te)

[Supporting figure 3A: LC-MS analysis of substrate Protocatechuate (PCA). 5](#_3znysh7)

[Supporting figure 3B: LC-MS analysis of PDC produced enzymatically via PmdAB and PmdC. 6](#_2et92p0)

[Supporting figure 3C: LC-MS analysis of CHMS produced enzymatically via PmdAB. 7](#_tyjcwt)

[Supporting figure 3D: LC/MS-MS analysis of CHMS elution peaks. 8](#_3dy6vkm)

[Supporting figure 4: Purification & crystallization of PmdC. 9](#_1t3h5sf)

[Supporting figure 5: Size-exclusion chromatography confirms PmdC is a dimer in solution. 9](#_4d34og8)

[Supporting figure 6: The three-dimensional structural similarity between PmdC and the Gfo/Idh/MocA superfamily 10](#_2s8eyo1)

[Supporting figure 7: Ligplot representation of interactions between NADP and PmdC 11](#_17dp8vu)

[Supporting figure 8: NADP binds PmdC within a tube-like cavity at the center of the protein molecule. 12](#_3rdcrjn)

[Supporting figure 9: Docking of CHMS substrate into PmdC – NADP structure. 13](#_26in1rg)

[Supporting figure 10: Sequence alignment of 18 CHMS dehydrogenases. 14](#_lnxbz9)

[Supporting Figure 11: Size exclusion chromatography of wild-type PmdC and H177A, H178A, H181A mutants 15](#_35nkun2)

[Supporting figure 12: QM optimized protonation states & bond distances (Å units) between CHMS and each active site histidine residue. 16](#_1ksv4uv)

[Supporting table 1: Bacterial strains and plasmids used in this study 17](#_44sinio)

[Supporting table 2: Primers used in this study 18](#_2jxsxqh)

[Supporting table 3: Comparison of kinetic parameters between PmdC, LigC and PmdAB, LigAB proteins 19](#_z337ya)

[Supporting table 4: QM calculated energy associated with each protonated form of histidine residues H177, H178 and H181 in the presence of modeled substrate and NADP. 20](#_3j2qqm3)

**Supporting figure 1: UV-Visible spectrum of PmdAB catalyzed conversion of PCA to CHMS**. The spectrum of 0.3 uM PCA alone at pH 7.5 is shown in transparent circles, the spectrum upon conversion of PCA to CHMS in the presence of 15 uM PmdAB at pH 7.5 is shown in black squares and the same CHMS spectrum in the presence of 3N NaOH is shown in black triangles. The CHMS spectral peak is at 410 nm.

**
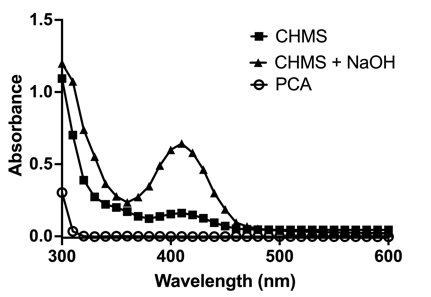
**

**Supporting figure 2:** **UV-Visible assays measuring the kinetics of PmdAB and PmdC.** (A) Kinetic data of the accumulation of CHMS over time at different concentrations of substrate PCA, measured by the increase in absorbance at 410 nm in the presence of PmdAB. (B) Kinetic data of the conversion of CHMS to PDC over time at different concentrations of substrate CHMS, measured by the decrease in absorbance at 410 nm in the presence of PmdC and NADP. (C) Kinetic data of the conversion of CHMS to PDC over time at different concentrations of substrate CHMS, measured by the decrease in absorbance at 410 nm in the presence of PmdC and NAD. (D) Negative control containing no cofactor. Each data point represents the mean and SD of three experiments.

**
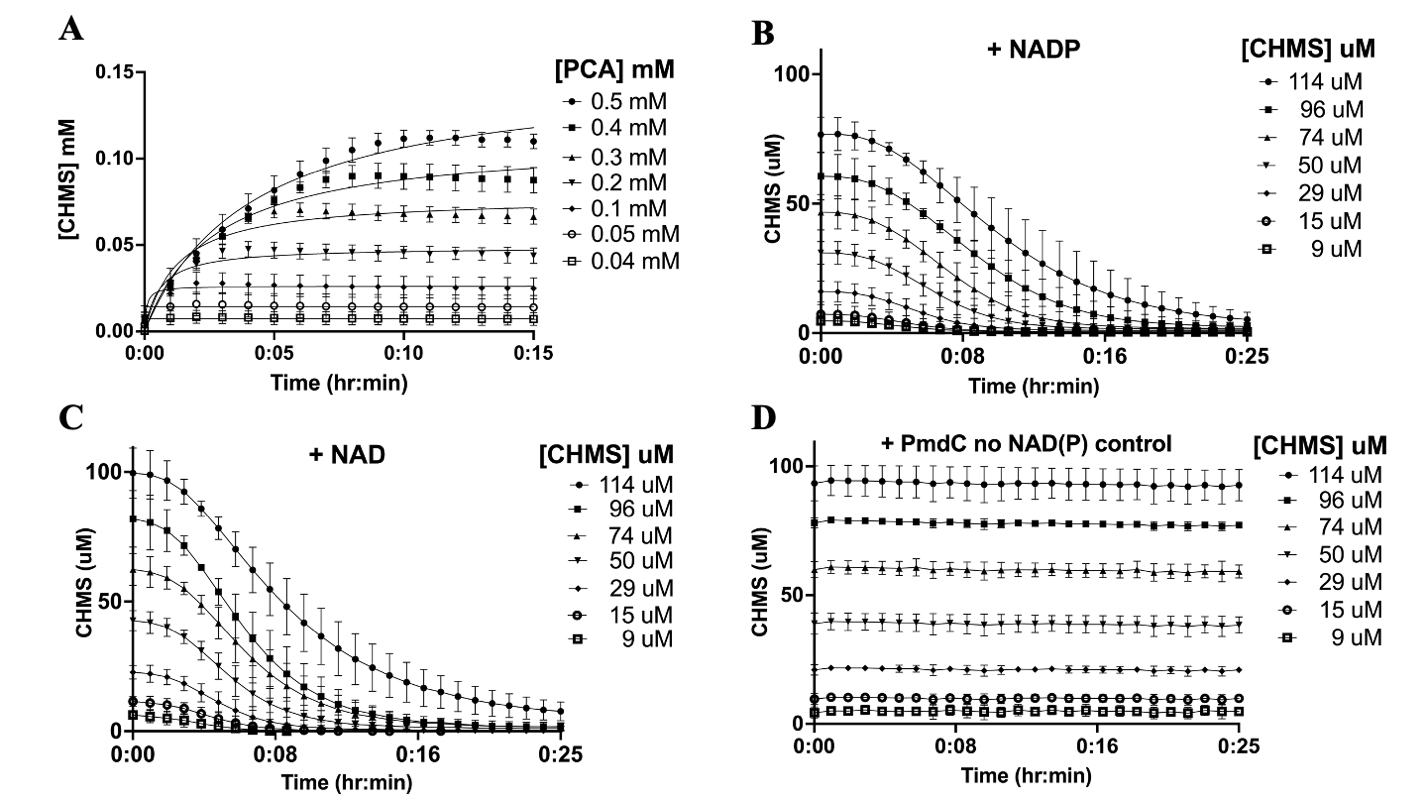
**

**Supporting figure 3A: LC-MS analysis of substrate Protocatechuate (PCA)**. EIC chromatograms of substrate PCA, inset: mass spectrum associated with the integrated peak. The upper panel corresponds to the experimental sample and the lower panel corresponds to a 20 uM standard. Observed mass error for all samples and standards are <5 ppm.



**
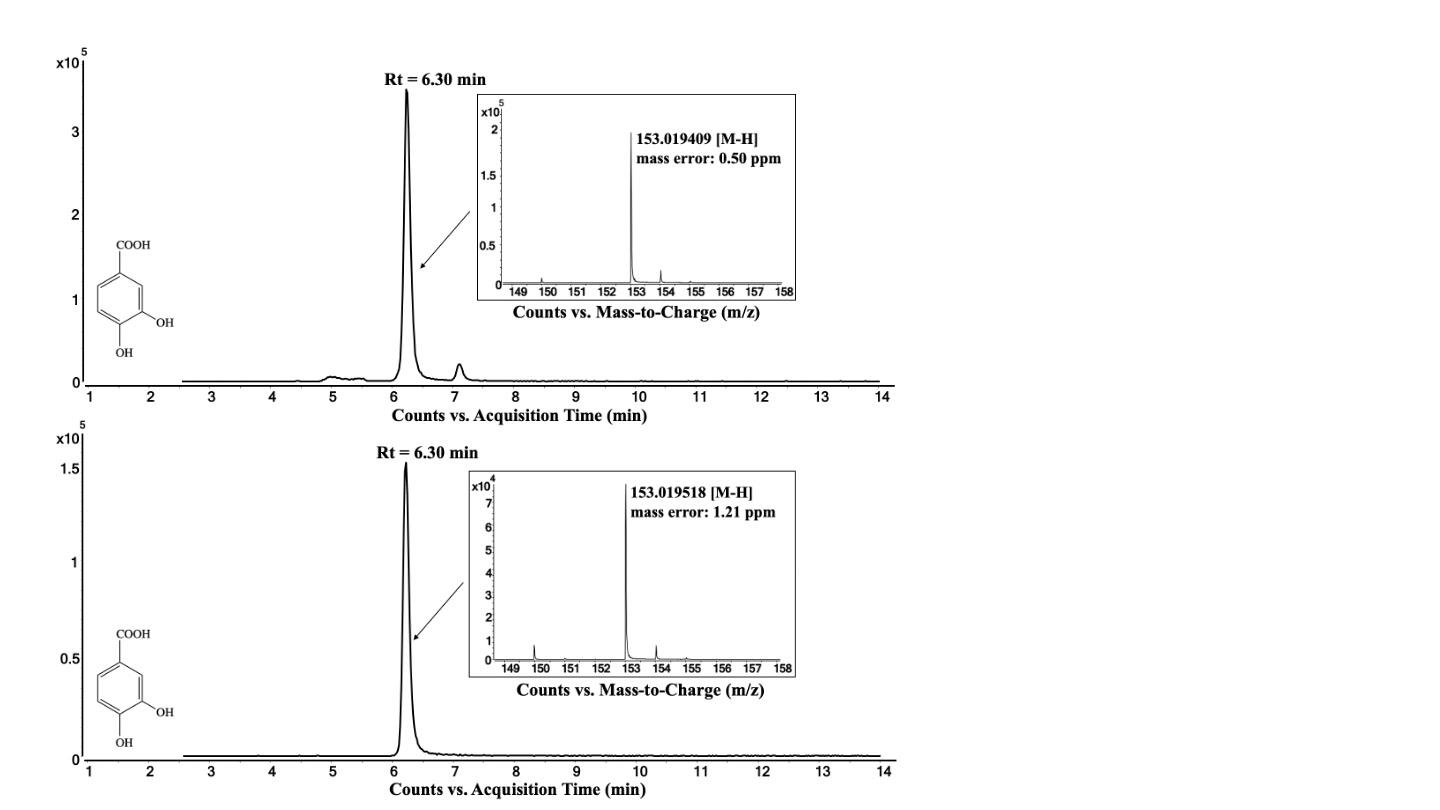
**

**Supporting figure 3B: LC-MS analysis of PDC produced enzymatically via PmdAB and PmdC.** EIC chromatograms of product PDC , inset: mass spectrum associated with the integrated peak. Upper panel corresponds to the experimental sample and the lower panel corresponds to a 20 uM standard. Observed mass error for all samples and standards are <5 ppm.



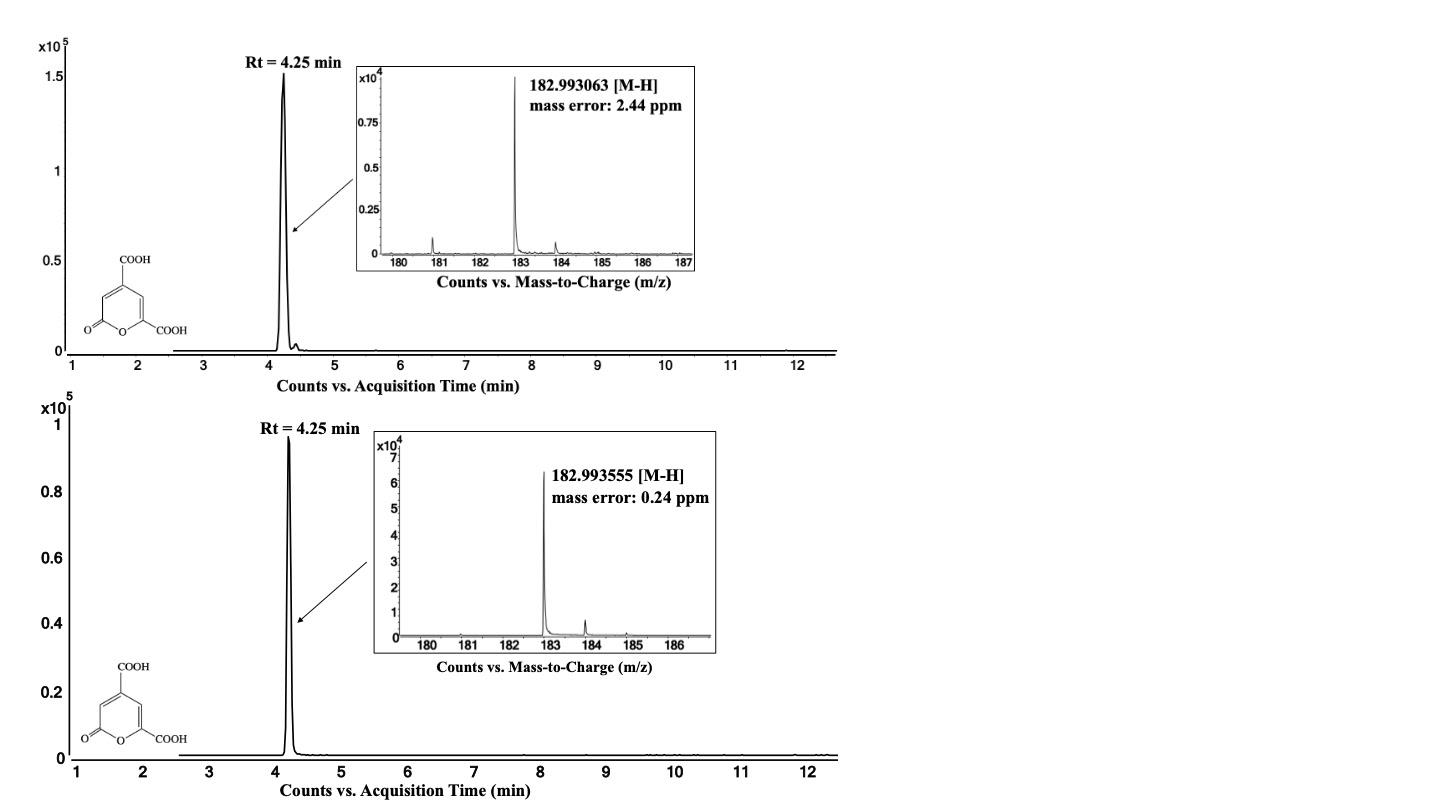


**Supporting figure 3C: LC-MS analysis of CHMS produced enzymatically via PmdAB.** EIC chromatogram of CHMS, inset: mass spectrum associated with the integrated peak. CHMS eluted as two separate peaks at retention times of 4.66 and 5.60 minutes. Fragmentation patterns obtained from LC-MS/MS for each peak were similar (Figure S3D). Observed mass error for all samples and standards are <5 ppm.



**
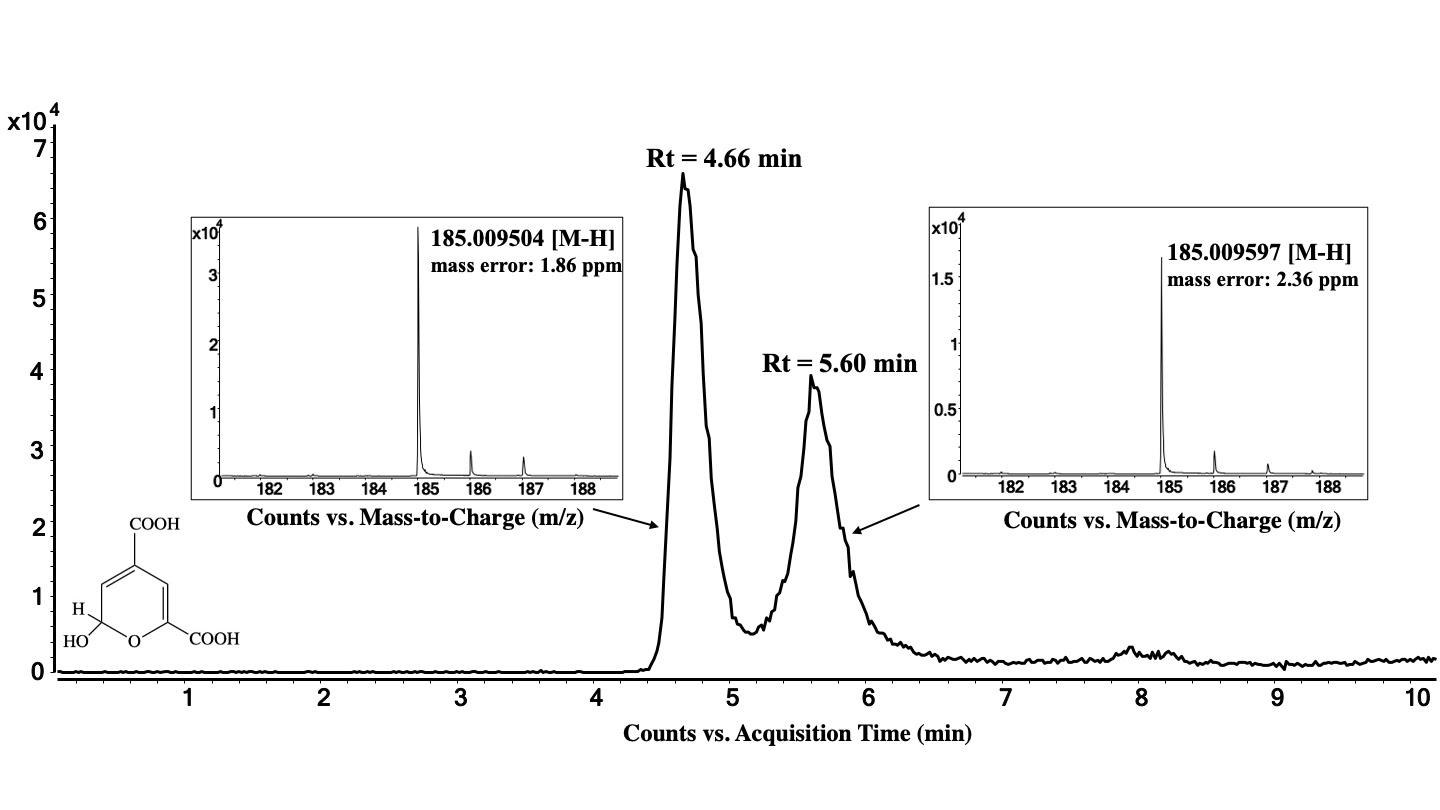
**

**Supporting figure 3D: LC/MS-MS analysis of CHMS elution peaks.** (A) A mass spectrum of the fragment ions from the first CHMS peak with a retention time of 4.66 min. The mass error associated with two fragment ions are m/z 69.035170 (8.43 ppm) and m/z 67.019049 (1.65 ppm) (B) A mass spectrum of the fragment ions from the second peak with a retention time of 5.60 min. The mass error associated with two fragment ions are m/z 69.034861 (3.95 ppm), and m/z 67.018609 (4.90 ppm). Higher mass errors were associated with ions at lower m/z, most likely due to the absence of internal reference/ lock mass ions (at <60 m/z) used to adjust the m/z axis in mass spectra post-data acquisition for improved mass accuracy. (C) Analysis of fragments ions arising from the hemiacetal and open-chain forms of CHMS, both forms yield fragments of similar m/z.

**
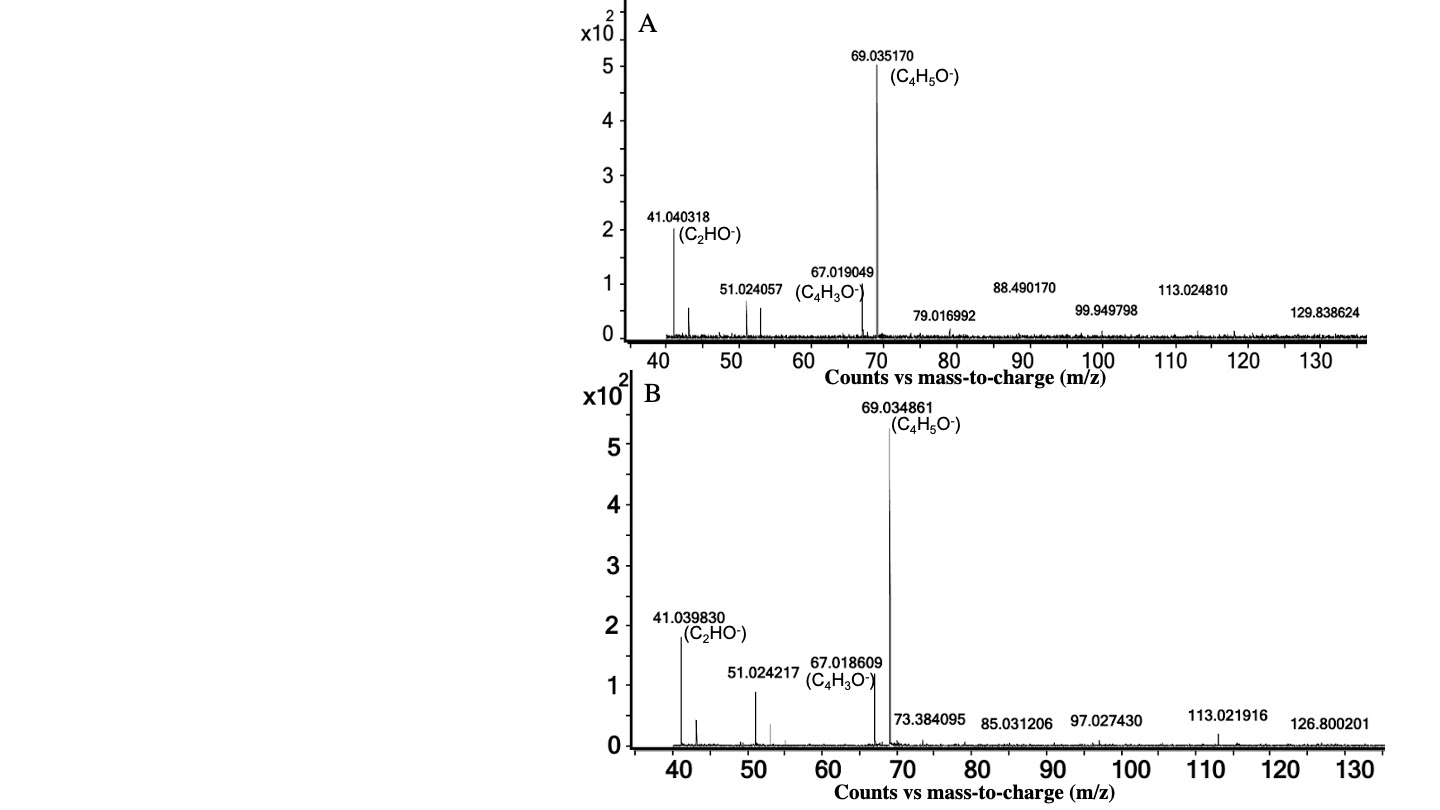
**

C

**
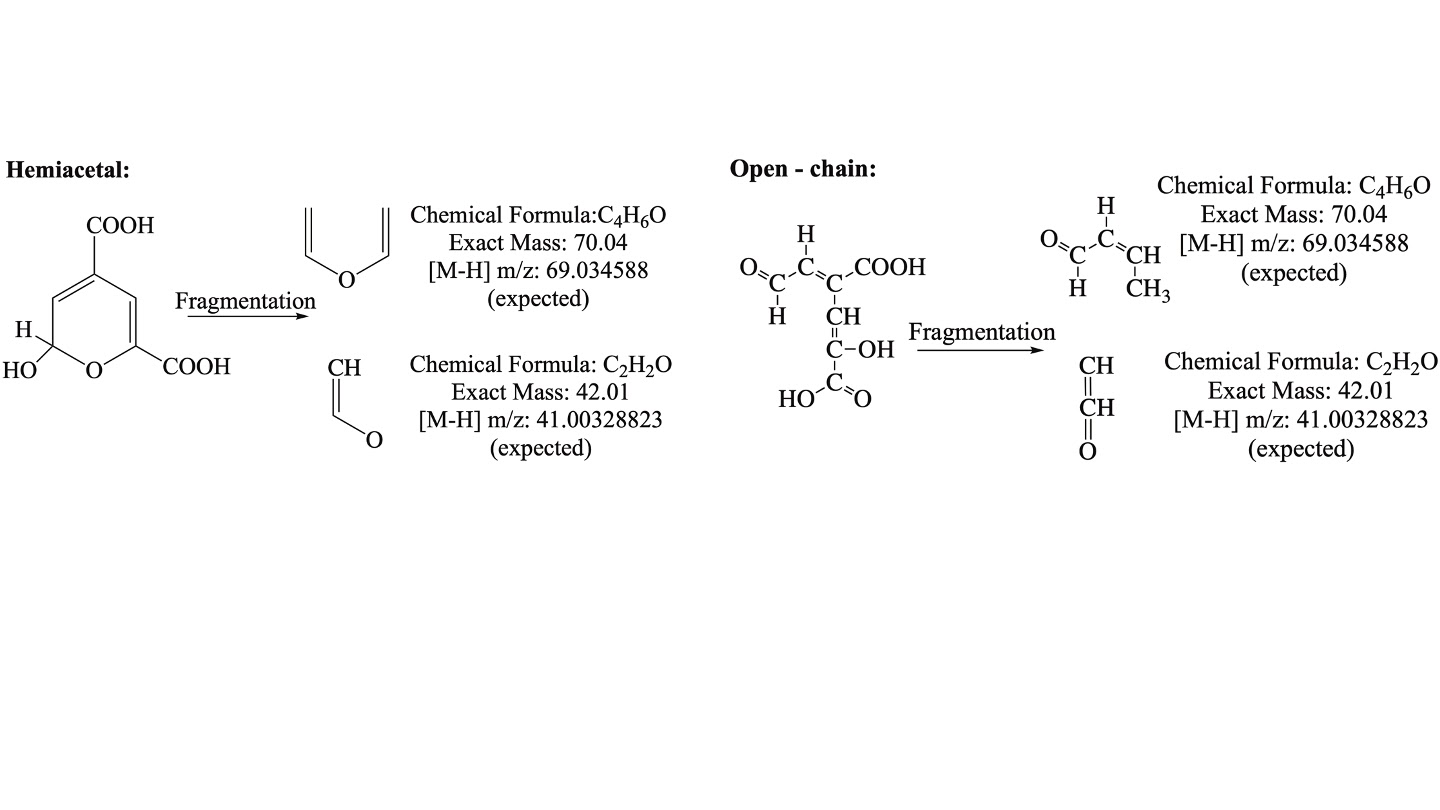
**

**Supporting figure 4: Purification & crystallization of PmdC.** (A) To assess the purity of PmdC, protein from the major size-exclusion chromatography (SEC) peak was visualized by SDS-PAGE (8-16% acrylamide tris/glycine SDS gel) and Coomassie blue staining. PmdC elution fractions were >95% pure and ran at a molecular weight (MW) of approximately 37 kDa. This protein was used for subsequent crystallization trials. (B) Plate type crystals of PmdC grown in 0.2M Ammonium Sulfate 0.1M Bis-Tris pH 5.5 25% w/v PEG 3350.

**A** **B**

**
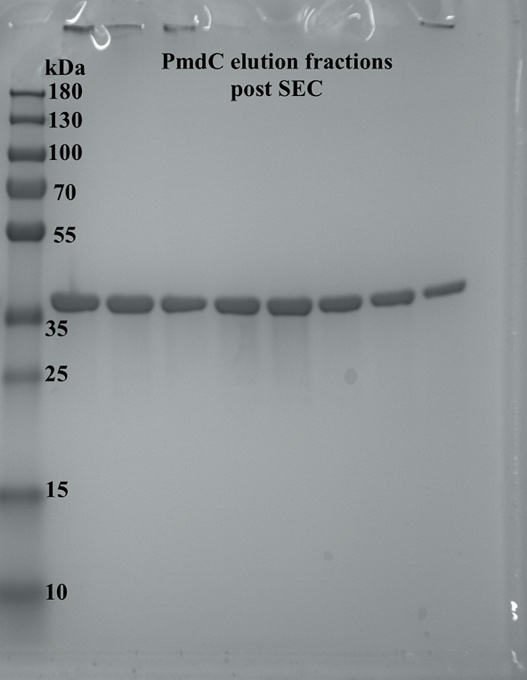

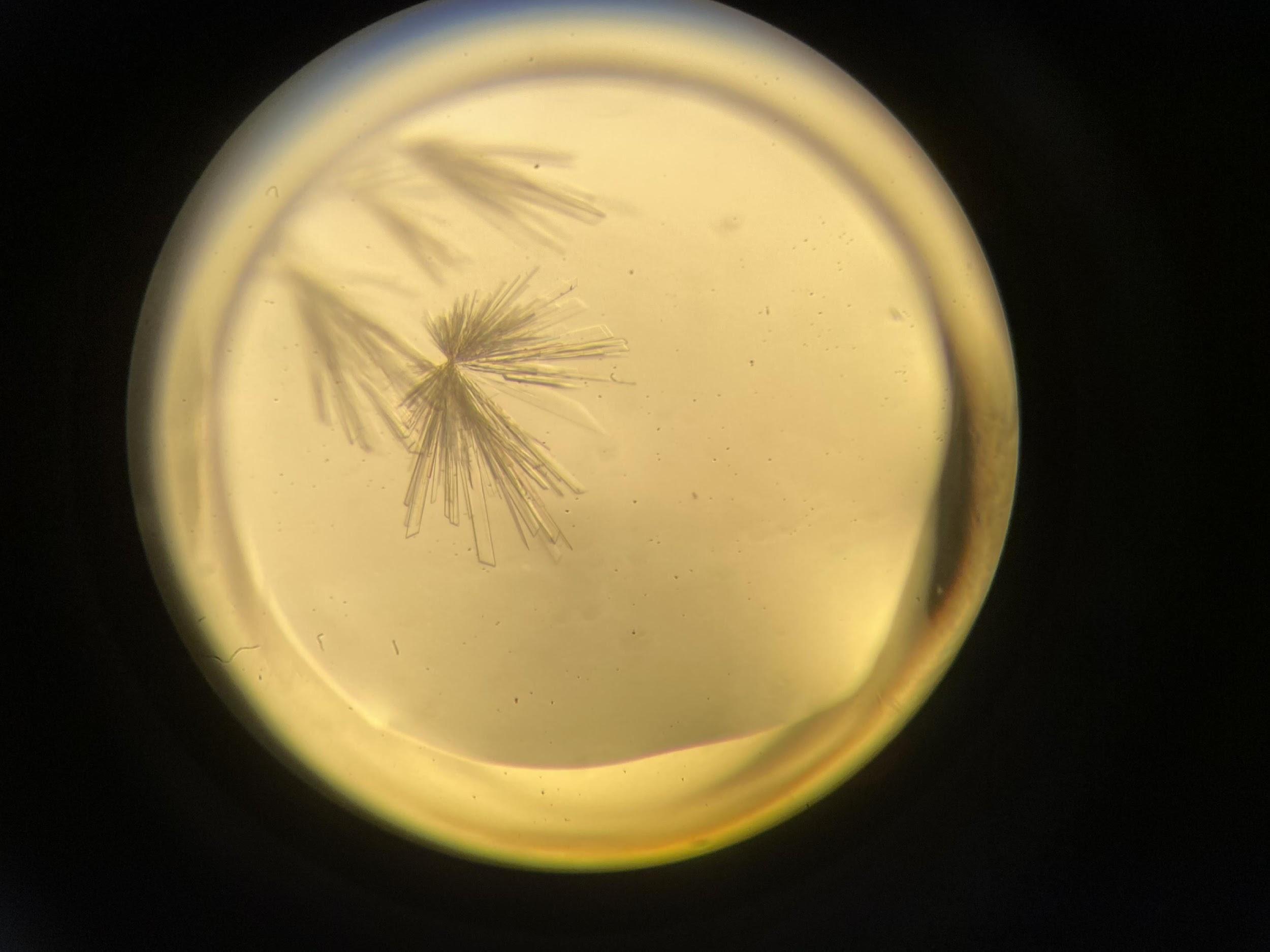
**

**Supporting figure 5: Size-exclusion chromatography confirms PmdC is a dimer in solution.** (A) Size-exclusion chromatography profile of PmdC showing a single peak at ~77.33 mL. This protein was used for crystallization and activity experiments. Inset, protein molecular weight calibration curve generated using Bio-rad protein standards of size (S1) 670 kDa, (S2) 158 kDa, (S3) 44 kDa, and (S4) 17 kDa . (B) Table shows the elution volumes of proteins that were run, in buffer containing 50 mM HEPES (pH 7.4), 150 mM NaCl, 1 mM DTT. PmdC displayed in red (calculated size ~70 kDa) eluted at a volume of 77.3 mL corresponding to a size consistent with being a functional dimer in solution.

**A** **B**

| **Proteins** | **Log_10_ (MW)** | **Integrated Peak Volume (mL)** |
| --- | --- | --- |
| Standard, S1 | 5.82 | 51.06 |
| Standard, S2 | 5.20 | 66.27 |
| Standard, S3 | 4.64 | 82.60 |
| Standard, S4 | 4.23 | 94.30 |
| PmdC | 4.84 | 77.33 |

**
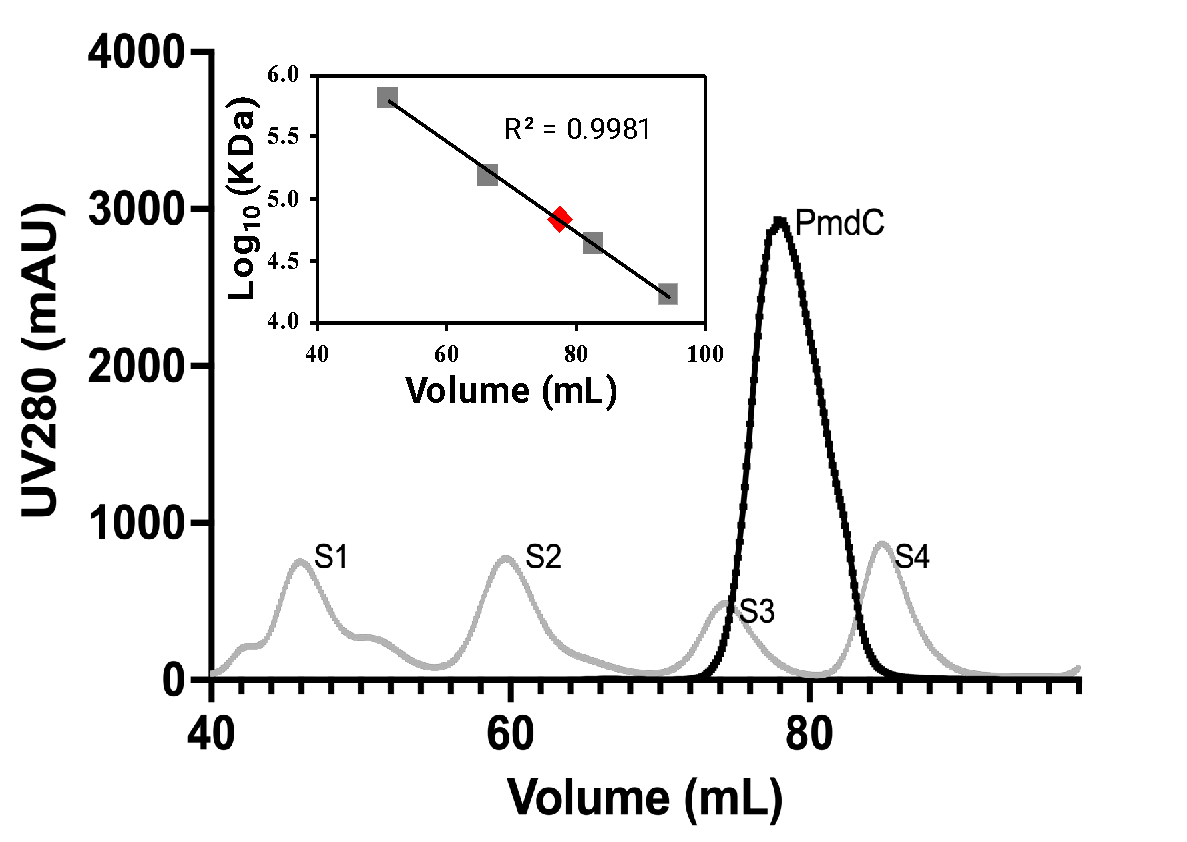
**

**Supporting figure 6: The three-dimensional structural similarity between PmdC and the Gfo/Idh/MocA superfamily.** (A-E) Comparison of the three dimensional structural features between (A) PmdC with (B) C-3''-ketoreductase (PDB 3RBV), (C) 1,5-anhydro-D-fructose reductase (PDB 4KOA), (D) Aldose-aldose oxidoreductase (PDB 5A02) and (D) Myo-inositol dehydrogenase (PDB 3NT2). Structures show a similar two domain fold, an N-terminal NAD(P) binding domain and the C-terminal substrate binding and oligomerization domain. (F) Three dimensional structures were aligned using PyMol, the structure of PmdC is shown in green. Superpositioning results (right table) show low RMSDs between PmdC and the structures indicating a very similar fold in spite of low sequence identity.


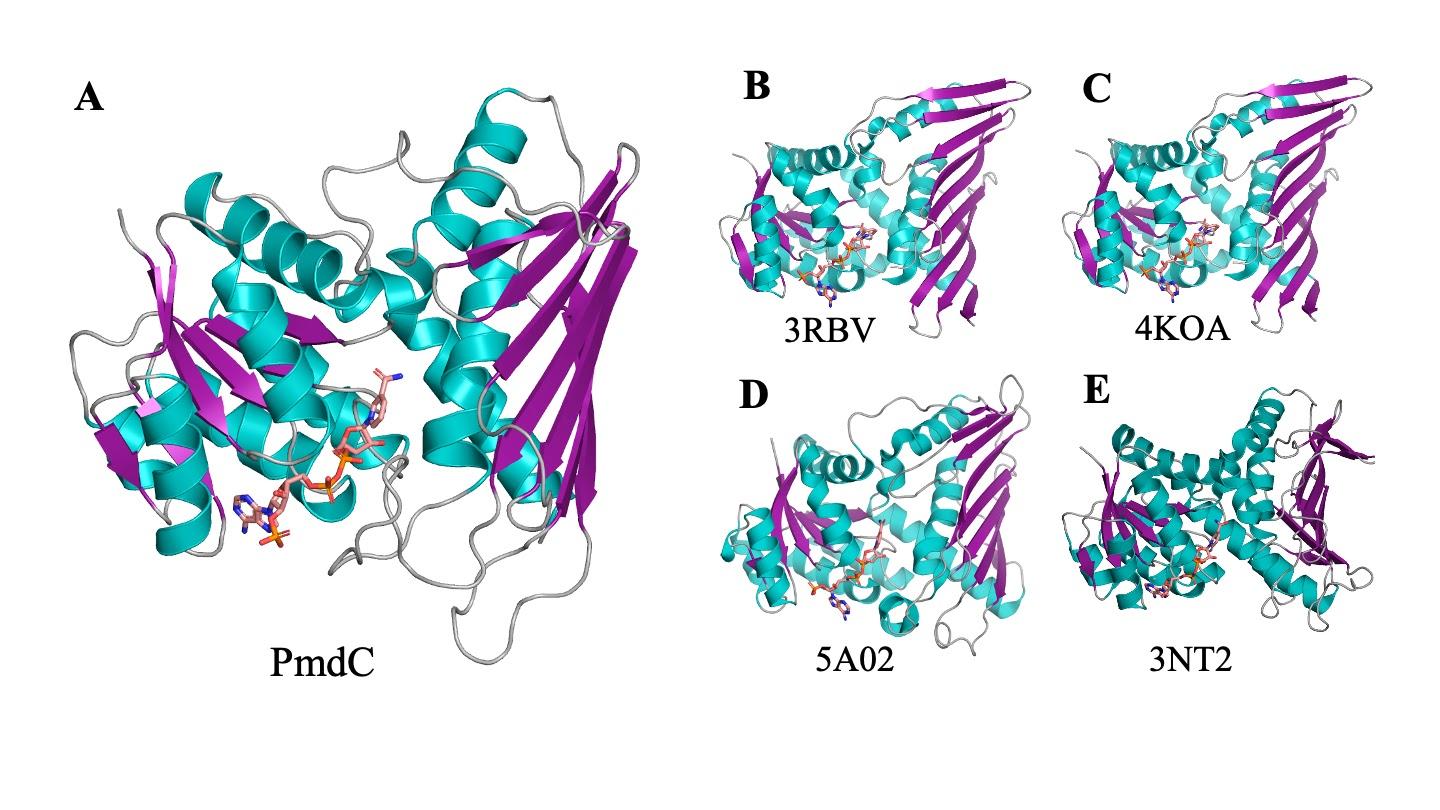


| **Protein** | **PDB ID** | | **PmdC alignment RMSD** |
| --- | --- | --- | --- |
| C-3''-ketoreductase | | 3RBV | 1.895 |
| 1,5-anhydro-D-fructose reductase | | 4KOA | 2.062 |
| Aldose-aldose oxidoreductase | | 5A02 | 1.870 |
| *Myo*-inositol dehydrogenase | | 3NT2 | 2.363 |

**
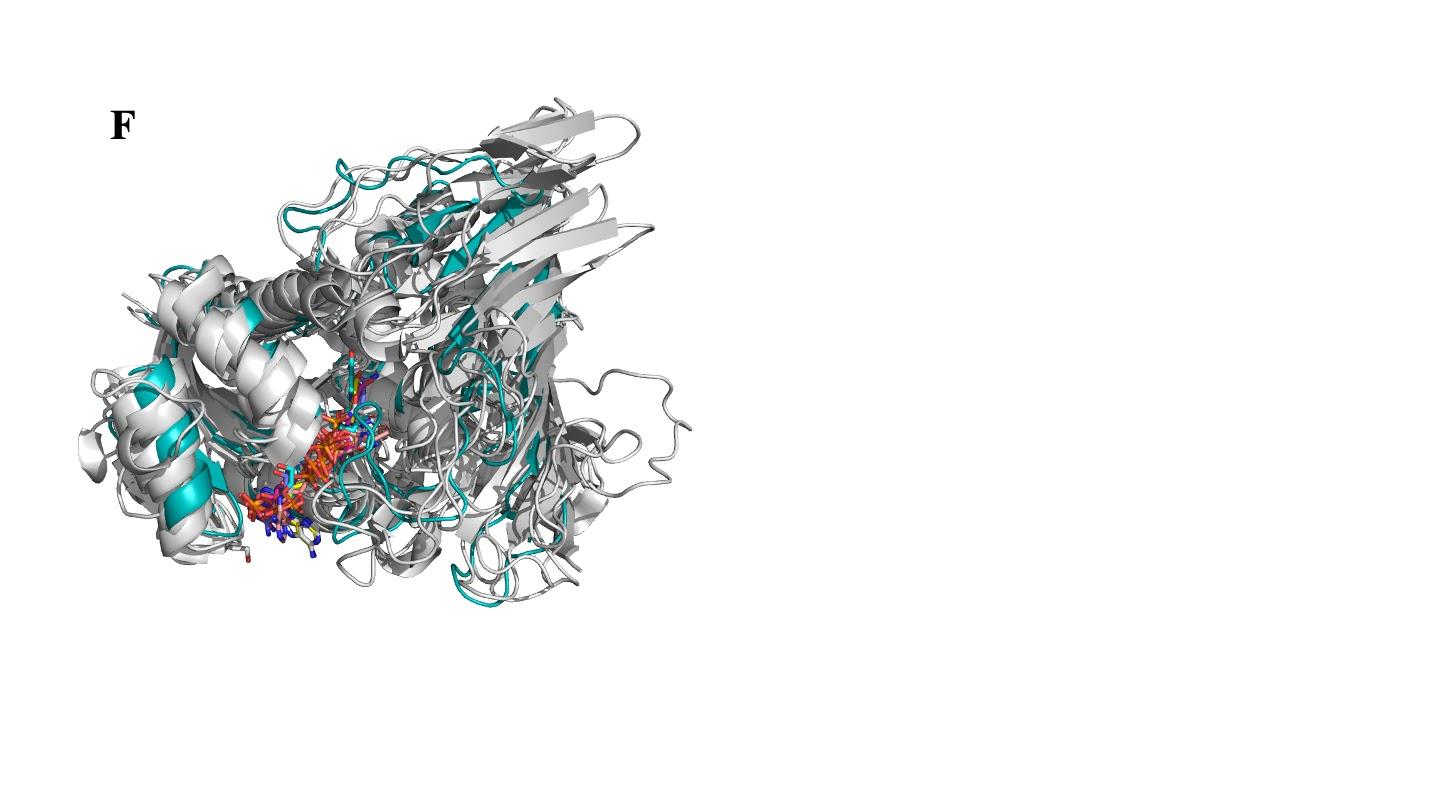
**

**Supporting figure 7: Ligplot representation of interactions between NADP and PmdC.** Hydrogen bond interactions between NADP (orange) and key residues (cyan) are displayed with dashed lines. Other non-bonded interactions are displayed by red spoked arcs.

**
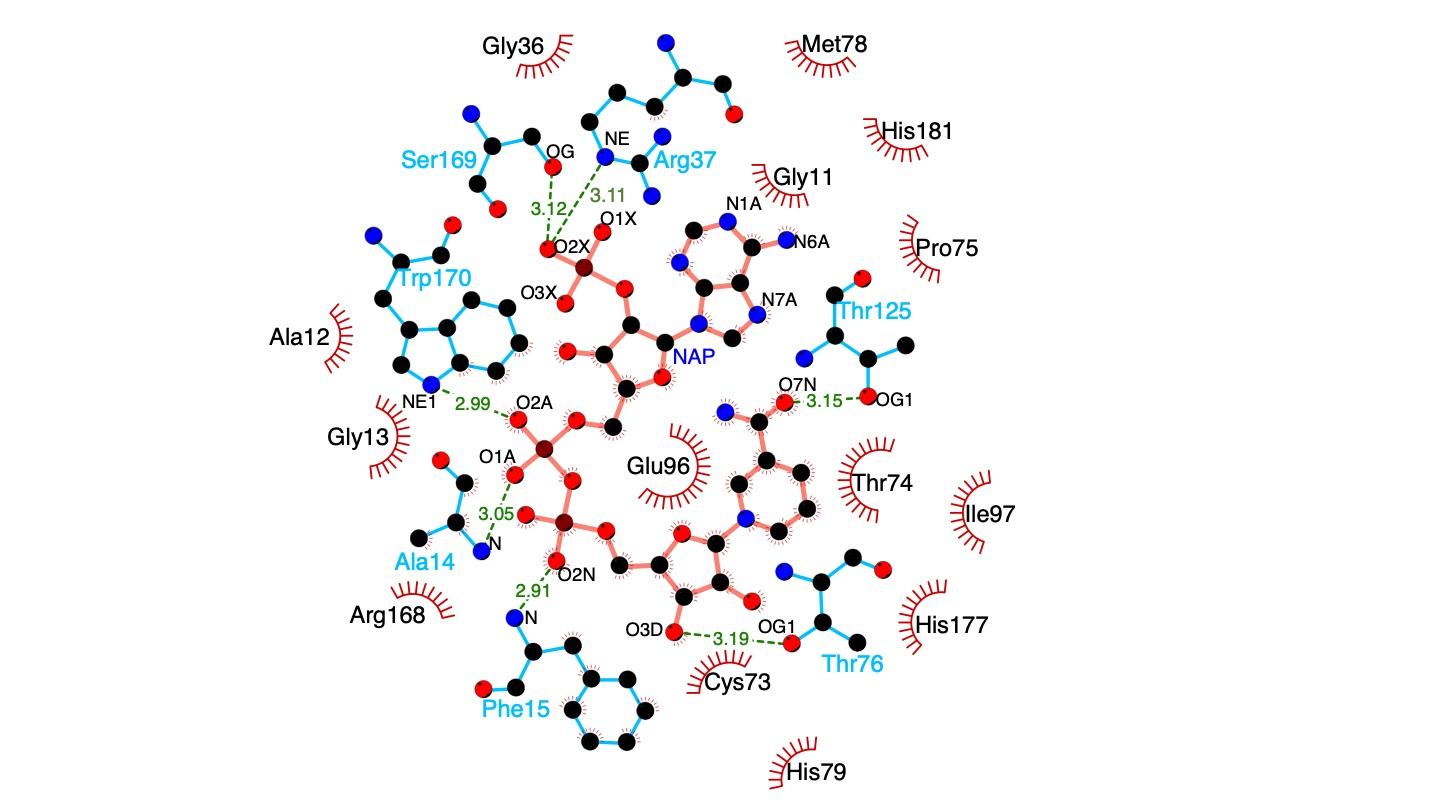
**

**Supporting figure 8: NADP binds PmdC within a tube-like cavity at the center of the protein molecule.** (A) Space filling model of the PmdC monomer with transparency set to 50% displaying the cartoon model bound to NADP within. The two openings within PmdC are marked by arrows, one at the initial βα loop at the N-terminal cofactor binding domain and the other at the middle of the molecule near the nicotinamide ring, possibly allowing substrate/product diffusion. Model was constructed using PyMol. (B) CASTp analysis (Tian et al., Nucleic Acids Res. 2018) of the PmdC cavity with openings designated by arrows. The cavity has a volume of 624 Å^3^ and was modeled using Chimera.

**
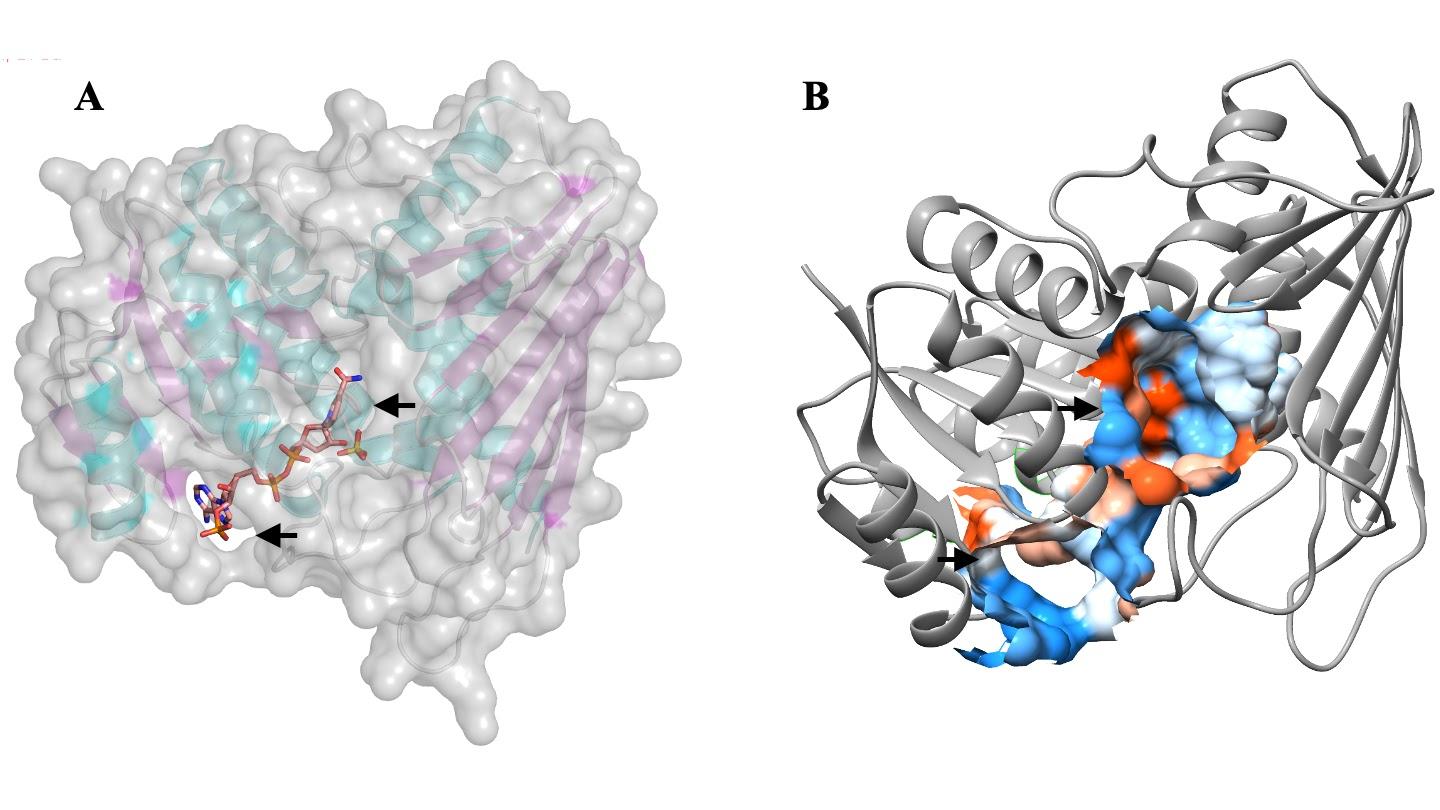
**

**Supporting figure 9: Docking of CHMS substrate into PmdC – NADP structure.** Lowest binding energy poses of CHMS modeled into the crystal structure of PmdC-NADP. Pose 1 in yellow had a binding energy of -5.08 kcal/mol and pose 2 in blue had a binding energy of -5.05 kcal/mol. After QM optimization, pose1 had 40 kcal/ mol lower energy compared to pose 2.

**
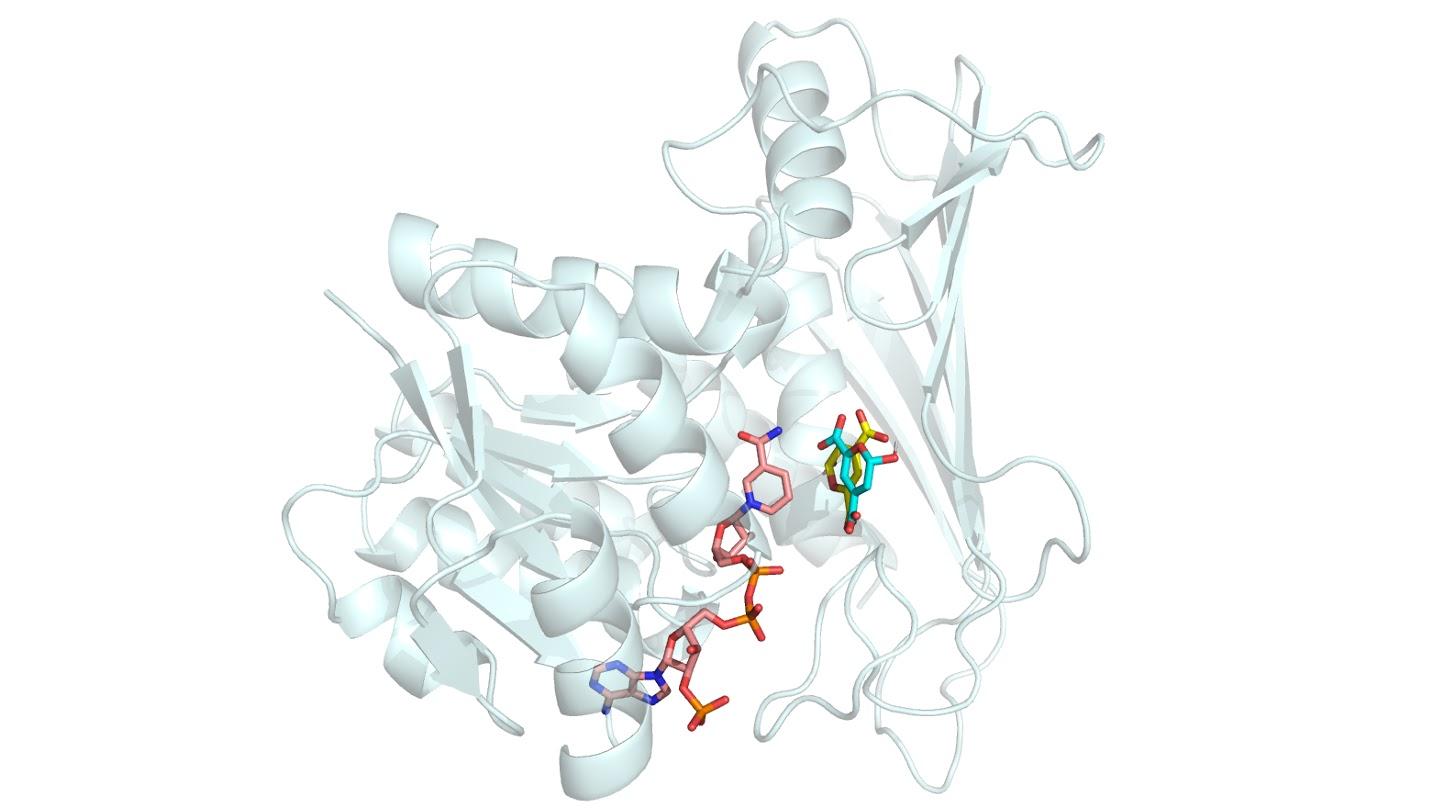
**

**Supporting figure 10: Docking of CHMS stereoisomers into PmdC – NADP structure. (A)** Binding energetics of stereoisomers of CHMS differing in spatial arrangement of the -OH and -H atoms at C2 position were analyzed by QMR. The (S)-2-hydroxy-2*H*-pyran-4,6-dicarboxylic acid isomer had a higher binding energy of -251.526 kcal/ mol while the (R)-isomer had a lower binding energy of -233.836 kcal/ mol. **(B)** Clashes (yellow line) were observed between the -OH group of the (R)-isomer and the nicotinamide ring. Clashes between CHMS and PmdC-NADP were analyzed using UCSF Chimera.

A

**
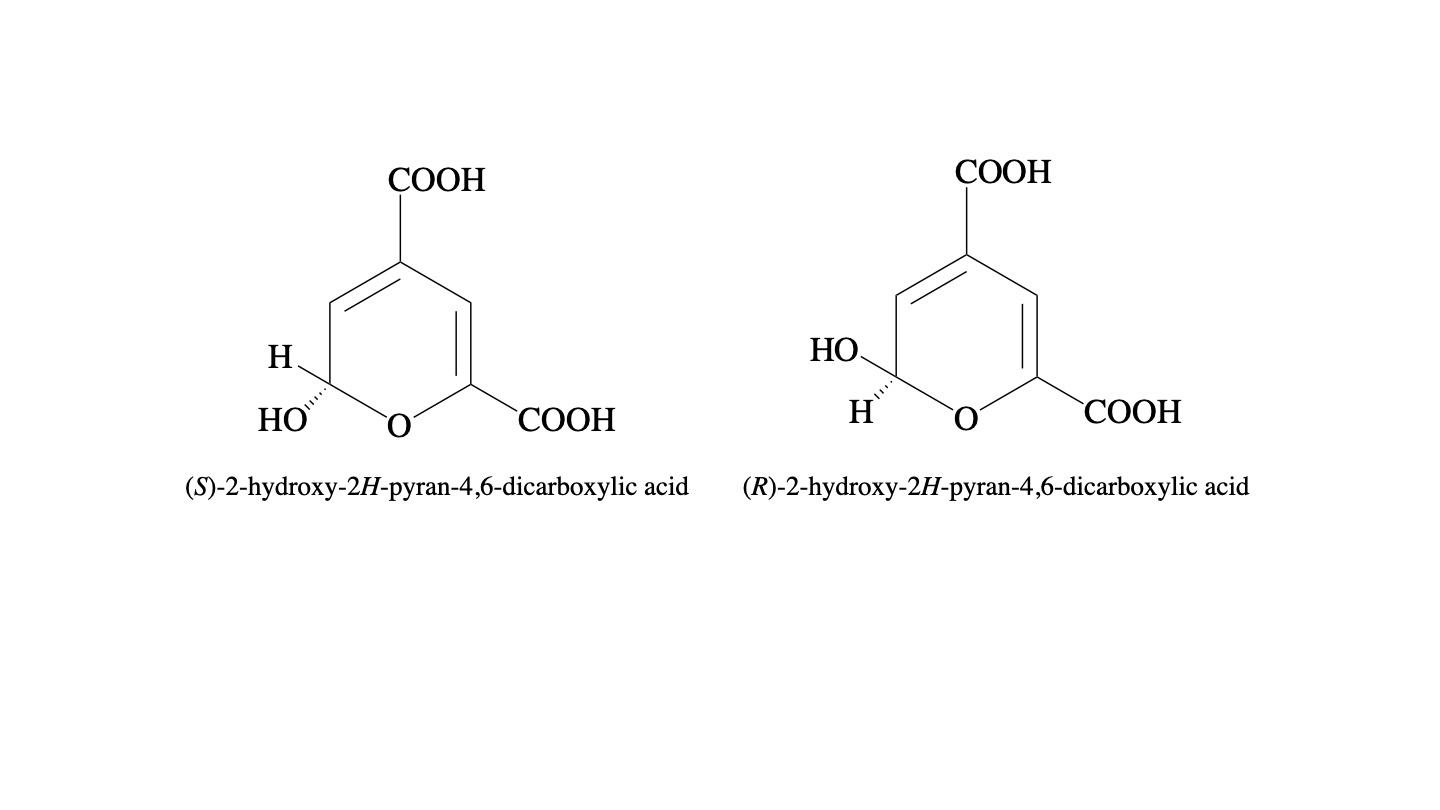
**

B

**
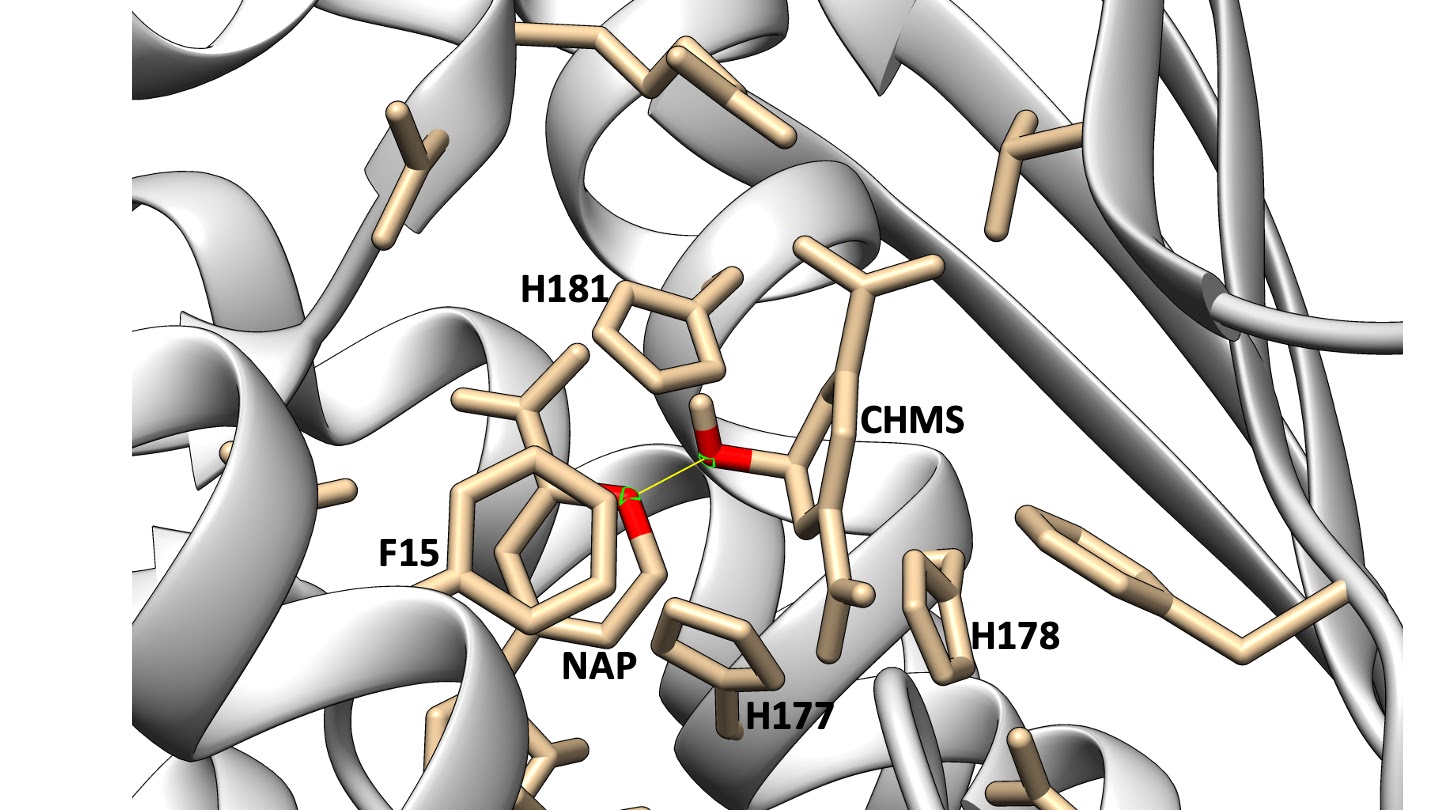
**

**Supporting figure 11: Sequence alignment of 18 CHMS dehydrogenases.** Aligned sequences show conserved residues involved in substrate binding, including the ^168^RSWTDHLLWHHXXHXXDXF^186^ sequence (PmdC numbering) containing HHXXH residues likely involved in catalysis. Homologs used in alignment were obtained from Zhou et al. (Biotechnology for Biofuels and Bioproducts, 2023). Species corresponding to each accession number and percent identity to PmdC are as follows: **AAK73574.1** (PmdC, *Comamonas testosteronii,* 100%); **AB073227.1** (LigC, *Sphingomonas* paucimobilis SYK-6, 76.11%); **AYG79828.1** (*Streptomyces hundungensis*, 69.91%); **NHO66817.1 (***Aestuariicella hydrocarbonica* JCM 30134, 79.68%); **KAB0542662.1 (***Kerstersia gyiorum* CCUG 47000, 83.02%), **AXF85169.1 (***Ephemeroptericola cinctiostellae* F02, 85.58%); **ACB35892.1 (***Leptothrix cholodnii* SP-6, 84.59%); **TCU95344.1 (***Pelomonas saccharophila* DSM 654, 80.88%); **MBQ0919763.1 (***Hydrogenophaga aromaticivorans* D2P3, 86.39%); **KQP37451.1 (***Pseudorhodoferax* sp. Leaf274, 86.39%); **SKA71468.1 (***Thiothrix eikelboomii* ATCC 49788, 69.43%); **EZP27613.1 (***Microbacterium oleivorans* RIT293, 65.82%); **MBE1527977.1 (***Sphingopyxis* sp. OAS728, 68.27%), **TIX48799.1 (***Alteraurantiacibacter aquimixticola* SSKS-13, 69.21%); **RIV77919.1 (***Pelagerythrobacter aerophilus* Ery1, 72.06%); **QWT16173.1 (***Sphingobium xenophagum* PH3-15, 75.08%); **MBB5734098.1 (***Altererythrobacter atlanticus* DSM 100738, 69.84%); **MBO9517657.1 (***Porphyrobacter* sp. BIN49, 74.52%).

**
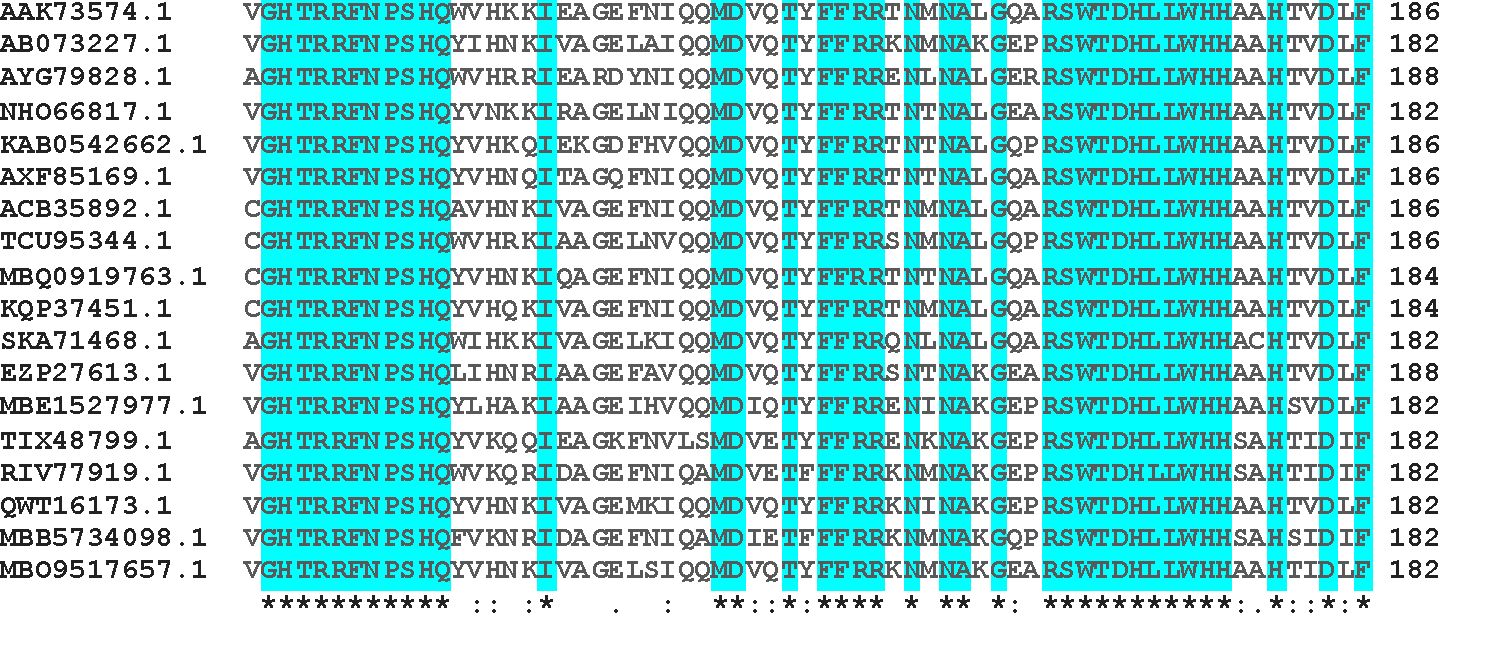
**

**Supporting Figure 12: Size exclusion chromatography of wild-type PmdC and H177A, H178A, H181A mutants.** Mutant proteins eluted at a volume of ~77.3 mL similar to wild-type, corresponding to a dimer in solution. These results indicate no significant alterations in structure. Inset: SDS-PAGE gel of wild-type PmdC and mutant proteins (conc. 100 μM) at a size of ~37 kDa.

**Supporting figure 13: QM optimized protonation states & bond distances (Å units) between CHMS and each active site histidine residue.** (A) H177, (B) H178 and (C) H181. Each residue is within hydrogen bond distance of the hydroxyl group of CHMS, these results together with site-directed mutagenesis studies indicate that the histidine residues play a role in substrate binding and catalysis.

**
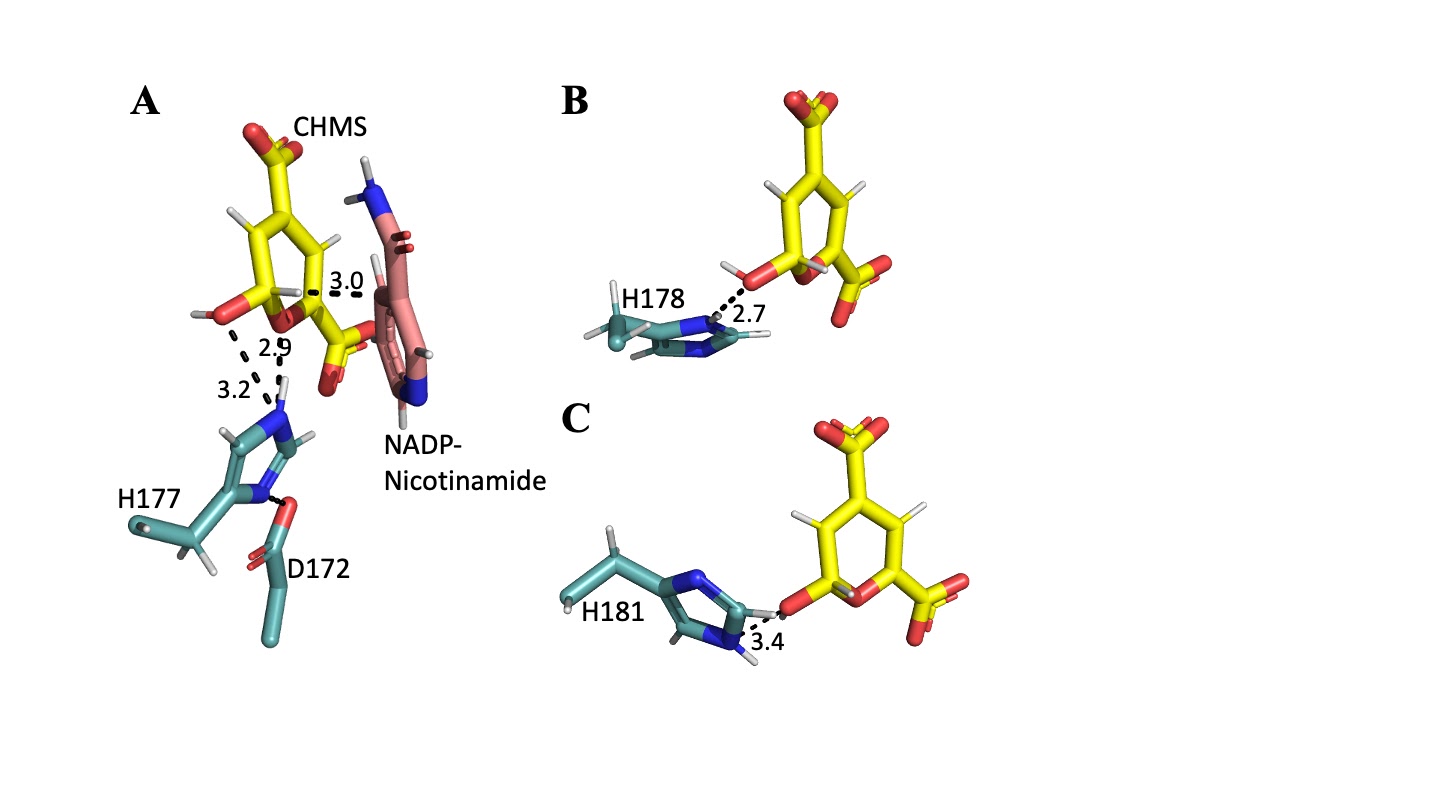
**

**Supporting Tables:**

## **Supporting table 1: Bacterial strains and plasmids used in this study**

| **Strain or plasmid** | **Relevant Characteristics** | | **Source or reference** | |
| --- | --- | --- | --- | --- |
| ***E. coli* strains:** |  | |  | |
| BL21 (DE3) | F^–^ *ompT* *gal* *dcm* *lon* *hsdSB* (r_B_^-^ m_B_^-^) λ(DE3) | | (Studier and Moffatt 1986) | |
| AR111 | BL21(DE3) with pAR111 | | (this study) | |
| AR211 | BL21(DE3) with pAR211 | | (this study) | |
| AR212  AR213  AR214 | BL21(DE3) with pAR212  BL21(DE3) with pAR213  BL21(DE3) with pAR214 | | (this study)  (this study)  (this study) | |
| **Plasmids:** |  | |  | |
| pAR111 | Km^r^; pSKB3 derivative vector wth ~1.4-kb fragment *pmdAB* cloned at NcoI and BamHI sites yielding a His6-PmdAB fusion protein with a TEV^a^ recognition site for His-tag removal. | | (this study) | |
| pAR211 | Km^r^; pSKB3 derivative vector with ~1-kb fragment of *pmdC* cloned between NcoI and BamHI sites yielding a His6-PmdC fusion protein with a TEV^a^ recognition site for His-tag removal. | | (this study) | |
| pAR212 | Km^r^; pSKB3 derivative vector with ~1-kb fragment of *pmdC* H177A cloned between NcoI and BamHI sites yielding a His6-PmdC H177A fusion protein with a TEV^a^ recognition site for His-tag removal. | | (this study) | |
| pAR213 | Km^r^; pSKB3 derivative vector with ~1-kb fragment of *pmdC* H178A cloned between NcoI and BamHI sites yielding a His6-PmdC H178A fusion protein with a TEV^a^ recognition site for His-tag removal. | | (this study) | |
| pAR214 | Km^r^; pSKB3 derivative vector with ~1-kb fragment of *pmdC* H181A cloned between NcoI and BamHI sites yielding a His6-PmdC H181A fusion protein with a TEV^a^ recognition site for His-tag removal. | | (this study) | |
| ^a^ TEV, Tobacco Etch Virus | |  | |  |

## **Supporting table 2: Primers used in this study**

| **Primer Name** | **Primer sequence** |
| --- | --- |
| ***For sequencing pmdC:*** |  |
| *pmdC_*Fwd | 5'-CTTGTTTGCATATCAGGCAGG-3' |
|  |  |
| ***For sequencing pmdAB:*** |  |
| *pmdAB_*Fwd | 5'-CAAAGGTTATGACTTCAGCC-3' |
| *pmdAB_*Rev | 5'-CAACAAGGAGTGGGACAAC-3' |
|  |  |
| ***pmdC* H177A mutant:** |  |
| *pmdC*_H177A_Fwd | 5’-CACTTGCTGTGGGCTCACGCTGCCCA-3’ |
| *pmdC*_H177A_Rev | 5’-TGGGCAGCGTGAGCCCACAGCAAGTG-3’ |
|  |  |
| ***pmdC* H178A mutant:** |  |
| *pmdC*_H178A_Fwd | 5’-GCTGTGGCATGCCGCTGCCCATACC-3’ |
| *pmdC*_H178A_Rev | 5’-GGTATGGGCAGCGGCATGCCACAGC-3’ |
|  |  |
| ***pmdC* H181A mutant:** |  |
| *pmdC*_H181A_Fwd | 5’-GCATCACGCTGCCGCTACCGTTGACTTG-3’ |
| *pmdC*_H181A_Rev | 5’-GGCAGCGTGAGCCCACAGCAAGTGATC-3’ |
|  |  |

## **Supporting table 3: Comparison of kinetic parameters between PmdC, LigC and PmdAB, LigAB proteins**

| **Protein** | **Substrate** | **Cofactor** | ***Km* (uM)** | ***Vmax* (µmole/min/mg)** |
| --- | --- | --- | --- | --- |
| PmdC (this study) | CHMS | NADP (1 mM) | 68.76 ± 2.4 (CHMS) | 450.06 ± 82.20 |
|  | CHMS | NAD (1 mM) | 62.70 ± 6.5 (CHMS) | 430.62 ± 143.45 |
| LigC (SYK-6)^1^ | CHMS (0.15 mM) | NADP | 24.60 ± 1.5 (NADP) | 449.0 ± 3.9 |
|  | CHMS (0.15 mM) | NAD | 252.0 ± 3.9 (NAD) | 363.0 ± 1.4 |
|  | CHMS | NADP (0.2 mM) | 26.00 ± 0.5 (CHMS) | 383.0 ± 3.8 |
|  | CHMS | NAD (0.2 mM) | 20.60 ± 1.0 (CHMS) | 175.0 ± 10.0 |
| LigC (P. ochraceae)^2^ | CHMS (0.16 mM) | NADP | 17.5 (NADP) | 470.0 |
|  | CHMS (0.16 mM) | NAD | 284 (NAD) | 470.0 |
|  | CHMS | NADP (0.11 mM) | 20.0 (CHMS) | 450.0 |
|  | CHMS | NAD (0.11 mM) | 56.0 (CHMS) | 167.0 |
| PmdAB (this study) | PCA |  | 33.0 ± 12.0 | 126.7 ± 14.5 |
| Lig AB (P.ochraceae)^1^ | PCA |  |  | 45.0 |

^1^ Masai et al., Journal of Bacteriology 2000

^2^ Maruyama et al., J. Biochem 1978

## **Supporting table 4: QM calculated energy associated with each protonated form of histidine residues H177, H178 and H181 in the presence of modeled substrate and NADP.**

|  | **H177** | | **H178** | | **H181** | |
| --- | --- | --- | --- | --- | --- | --- |
| **Histidine protonation site** | **Energy (Hartree units)** | **Energy (kcal/mol)** | **Energy (Hartree units)** | **Energy (kcal/mol)** | **Energy (Hartree units)** | **Energy (kcal/mol)** |
| **ND1, NE2** | -364.80136 | 36.33 | -451.75504 | 90.47 | -384.40842 | 110.22 |
| **ND1 only** | -364.85210 | 4.49 | -451.88675 | 7.81 | -384.56019 | 14.99 |
| **NE2 only** | -364.85926 | 0.00 | -451.89865 | 0.35 | -384.58407 | 0.00 |
| **ND1, NE2 flipped** | -364.80412 | 34.60 | -451.75501 | 90.48 | -384.41922 | 103.44 |
| **ND1 only flipped** | -364.85826 | 0.63 | -451.89920 | 0.00 | -384.57726 | 4.27 |
| **NE2 only flipped** | -364.85881 | 0.28 | -451.88693 | 7.70 | -384.57575 | 5.22 |
